# Peer Relationships Are a Direct Cause of the Adolescent Mental Health Crisis: Interpretable Machine Learning Analysis of 2 Large Cohort Studies

**DOI:** 10.2196/60125

**Published:** 2025-05-12

**Authors:** Heiner Stuke, Robert Schlack, Michael Erhart, Anne Kaman, Ulrike Ravens-Sieberer, Christopher Irrgang

**Affiliations:** 1 Centre for Artificial Intelligence in Public Health Research of the Robert Koch-Institute, Nordufer 20, Berlin, 13353, Germany, 49 30 18754 211; 2 Department of Psychiatry and Neurosciences at the Charité Campus Mitte, Charité University Hospital Berlin, Berlin, Germany; 3 Department B Epidemiology and Health Monitoring, Robert Koch-Institute, Berlin, Germany; 4 Department of Child and Adolescent Psychiatry and Psychotherapy and Psychosomatics, University Medical Center Hamburg-Eppendorf, Hamburg, Germany

**Keywords:** mental health, depression, epidemiology, machine learning, prediction, risk factors, adolescence, adolescent, adolescent health, peer, health crisis, Western countries, longitudinal study, cohort study, screen time, physical inactivity, social isolation, public health, assessments, analogous analyses, Pearson correlation

## Abstract

**Background:**

Converging evidence indicates an adolescent mental health crisis in Western societies that has developed and exacerbated over the past decade. The proposed driving factors of this trend include more screen time, physical inactivity, and social isolation, but their causal influence on mental health is insufficiently understood.

**Objective:**

The objective of this study is to test whether and based on which predictor variables the development of mental health in adolescents in the last decade can be predicted and to better understand the causal chain of factors at work.

**Methods:**

We implemented an interpretable machine learning pipeline based on gradient boosting regression with repeated cross-validation to assess the development of mental health throughout adolescence in members of 2 longitudinal cohort studies, the British Millenium cohort (MC; n=8599) and the German Health Interview and Examination Survey for Children and Adolescents (KiGGS) cohort (n=1212). In total, 144 (MC) and 102 (KiGGS) predictors assessed at the age of around 13.8 years (MC) and 11.6 years (KiGGS) were used to assess mental health at the ages of around 16.7 years (MC) and 16.4 years (KiGGS). Based on these predictive models, we used permutation-based feature importance analyses to identify relevant predictors and predictor domains. Moreover, we performed partial dependence analyses in a causal inference framework to determine the direct effects of physical inactivity, screen time, and peer problems on the development of mental health.

**Results:**

The average cross-validated Pearson correlation coefficient (*r*) between predicted and true mental health in late adolescence was 0.614 (MC) and 0.466 (KiGGS). Feature importance analyses indicated a strong impact of preexisting mental health and weaker impacts of sex (female as a risk factor), physical health (chronic disease as a risk factor), lifestyle, and socioeconomic and family factors (eg, low parental education, income, and mental health as risk factors). Causal inference analyses suggested a strong direct effect of peer relationships, but only a small direct effect of physical inactivity and a very small direct effect of screen time.

**Conclusions:**

Mental health development during adolescence can be assessed by a combination of variables from early adolescence. Peer problems represent an important direct cause of mental health development, and their deterioration may contribute to the current mental health crisis.

## Introduction

Adolescence is a critical period of life when people explore, experiment, and are exposed to particular risk factors such as substance use, violence, bullying, and academic problems. It is when the course is set for health in adulthood [1]. The current debate about an adolescent and young adult mental health crisis in Western countries [2,3] is therefore all the more important, especially because it is substantiated by a number of recent findings: a study comparing 2 UK birth cohorts found that while no difference was found during childhood, there were markedly more emotional problems during adolescence in individuals born 2000‐2002 compared to individuals born 1991‐1992 [4]. Consistently, a recent comparison of 2 methodologically standardized and comparable Dutch population surveys showed almost a doubling of affective disorders between the years around 2008 and around 2021, with the increase being particularly pronounced among young adults [5]. In the United States, after a years-long decline, rising depression among adolescents has been reported since about 2010 [6], accompanied by an increase in suicide rates in this age group [7]. While prevalence rates for externalizing mental health problems among children and adolescents in Germany were declining before the COVID-19 pandemic, emotional problems also increased here between 2003‐2006 and 2014‐2017, especially among girls [8]. These alarming results are in line with findings from other countries, although there is heterogeneity and effect sizes appear slightly smaller in meta-analytic summaries [9]. While reduced stigma and an increased readiness to report mental health problems might partly contribute to these trends, it is remarkable that rising prevalence rates are also reported in studies that used the same standardized and well-validated clinical interview over different time periods [5]. Moreover, investigations relating time trends of self-reported mental disorders to time trends of stigmatization of mental disorders in the same regions did not find evidence that reduced stigma explains the increase in self-reported mental health problems [10].

Summarizing the evidence suggests that since about 2010, there seems to be a marked cross-national increase in mental health problems of adolescents, particularly concerning internalizing or emotional problems and affective disorders. The causes for this increase are insufficiently understood [2]. Proposed risk factors cover rising screen time and social media consumption [11], worsened peer relationships with loneliness and social isolation [12], and physical inactivity [13]. However, the predictive strength and the causal interplay of these factors are still unclear and debated.

To address this research gap, we used multivariable machine learning models to assess the development of mental health from early to late adolescence in members of longitudinal cohort studies. This allows us to model nonlinear relationships between numerous predictors in early adolescence and the future development of mental health, as well as to test the quality of the resulting predictions by means of cross-validation. We investigated 2 large independent population-based cohort studies of adolescents, the UK Millennium Cohort (MC) study [14] and the German Health Interview and Examination Survey for Children and Adolescents (KiGGS) [15]. This approach allows a robust comparison of results between 2 independent cohorts from different countries.

The analyzed cohort studies were conducted in countries affected by the mental health crisis, and the investigated time period covers exactly the “critical period” of the crisis development from 2010 onwards. Thus, the developed models can also be used to add further evidence to the debate on driving factors of this deterioration. More specifically, it can be examined which simulated changes in risk factors that have been implicated in the ongoing mental health crisis actually led to worse predicted mental health. However, assessing beyond pure predictive relationships the causal impact of these risk factors on the development of mental health involves stronger assumptions and challenges interpretation [16]. In this work, we draw on our multivariable models in conjunction with recent research on the causal interpretation of predictive models [17] and on the appropriate selection of covariates [18]. Based on this approach, we assess the independent effect of proposed driving factors for the adolescent mental health crisis (increased screen time, less physical activity, and worsened peer relationships).

We aimed to answer 3 related research questions. (1) How well can mental health in late adolescence be assessed based on assessments in early adolescence? (2) Which assessments in early adolescence are important for these assessments? (3) What are the independent impacts of physical activity, screen time, and peer relationships on unfavorable mental health trajectories of adolescents in the years after 2010?

## Methods

### Analyzed Cohort Studies

For reciprocal validation, analogous analyses were conducted in 2 European cohort studies of child and adolescent health. The first one was the MC study investigates the development of children born in 2000-2002 in the United Kingdom [19]. For this study, we assessed the participants’ mental health at the age of around 17 (hereafter referred to as “follow-up acquisition”) based on assessments at the age of around 14 (“baseline acquisition”). The second one was the KiGGS, a population-based study for German children and adolescents with 3 longitudinal assessment waves [15]. In KiGGS, we used participants’ assessments at the age of around 12 years (“Baseline acquisition”) to assess their mental health at the age of around 16 years (“Follow-up acquisition”). A participant flowchart for both cohorts is shown in Figure S1 in Multimedia Appendix 1, and the final sample is described in Table 1.

**Table 1.** Descriptive statistics summary of cohort participants.

	Millennium cohort	KiGGS^a^ cohort
Number of eligible participants (m/f), n (n/n)	8599 (4268/4331)	1212 (599/613)
Period of baseline acquisition	January 2015 to March 2016	June 2009 to June 2012
Period of follow-up acquisition	January 2018 to March 2019	September 2014 to August 2017
Age at baseline (years), mean (SD)	13.76 (0.46)	11.62 (0.62)
Age at follow-up (years), mean (SD)	16.73 (0.46)	16.37 (0.65)
Total number of predictors at baseline, n	144	102

a KiGGS: German Health Interview and Examination Survey for Children and Adolescents.

### Ethical Considerations

All MC waves were approved by medical research ethics committees, specifically by the National Research Ethics Service Research Ethics Committee London—Central for the baseline acquisition (approval 13/LO/1786) and by the National Research Ethics Service Research Ethics Committee North East—York for the follow-up acquisition (approval 17/NE/0341). The ethics committee of the Charité – Universitätsmedizin Berlin has approved the KiGGS baseline study (approval 101/2000) and KiGGS Wave 1 (approval EA2/058/09), and the ethics committee of the Hannover Medical School has approved KiGGS Wave 2 (approval 2275‐2014).

### Outcome, Predictors, and Sample Weights

Consistent with previous studies [4] and because of its availability in both cohorts at both examination times, we used the emotional symptoms sum score of the parents-reported Strength and Difficulties Questionnaire (SDQ) as the outcome measure in both cohorts. The SDQ is among the most established assessment tools for mental health in children and adolescents [20]. Its emotional symptoms subscale (SDQ-E) consists of 5 items on a 0‐2 Likert scale, which are summed to give a total score ranging from 0 to 10. It has demonstrated a high accuracy in detecting depressive and anxiety disorders [21].

Based on consultations with experts in adolescent mental health (RS, URS), the study assessments were systematically screened and a broad set of predictors of future mental health was included and assigned to the domains of mental health and well-being (eg, questions on depressive and anxiety symptoms, history of self-harm [22]), physical health (chronic diseases, BMI [23]), psychological and cognitive testing (school grades, cognitive assessments, moral attitudes), socioeconomic and family factors (family climate, socioeconomic status, and mental health problems of the parents [24,25]), lifestyle and peer relationships (substance use, media consumption, activities [11,26,27]), and others (sex [28]). A complete list of selected predictors, domain assignments, and the percentage of missing values can be found in Tables S1 and S2 in Multimedia Appendix 1. The analysis of predictor domains in addition to individual predictors aimed to simplify the interpretation of the feature importance analyses and to stabilize the results against the inclusion or exclusion of individual predictors. Hence, in contrast to data-driven dimension reduction techniques, the focus here was not on an explanation of the highest possible variance of the predictors by the factors but on their conceptual interpretability. However, post-hoc tests showed that these content-based domain assignments significantly better captured the latent factor structure of the data as compared to random assignments (Text S1 in Multimedia Appendix 1).

Both investigated cohort studies aimed for representativeness for the total population of the surveyed country. However, because individuals with certain sociodemographic criteria were underrepresented in the sampled cohort compared with the total population, both cohorts provide weight variables to give individuals from underrepresented groups more weight in the analyses. To further increase the generalizability of our results to the overall country population, we used these weights (GOVWT2 variable for the MC, wQSpop variable for KiGGS) in all univariable and multivariable analyses.

### Univariable Comparisons

To obtain unadjusted associations between individual predictors and outcomes, we calculated linear models with each predictor as the only independent variable, based on data completed using K-nearest neighbors imputation (KNNImputer class of the scikit-learn toolbox for Python [29] with default parameters) and weighted with population sample weights. Exploratory tests on the quality of the values imputed in this way revealed highly significant correlations between imputed and actual values, which would not be achievable with univariate imputation methods such as mean or median imputation (Text S2 in Multimedia Appendix 1). Here, numerical predictors were z-standardized (so that the resulting coefficient values are identical to Pearson correlation coefficients). These analyses make it possible to assess correlative (uncorrected) relationships between the individual predictors of interest and mental health in later adolescence.

### Multivariable Machine Learning Pipelines

**Figure 1.**
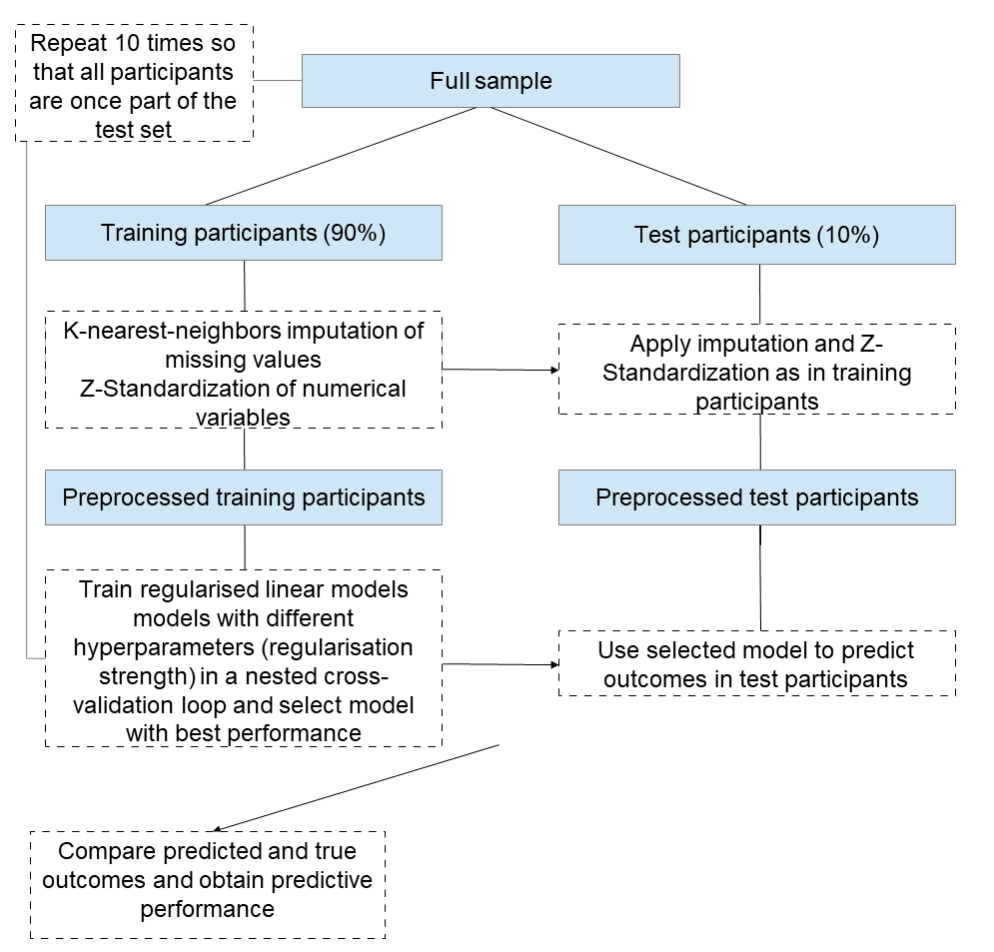
The linear pipeline comprised one-hot encoding of categorical variables, K-nearest neighbor imputation of missing values (as implemented in the scikit-learn KNNImputer class with default parameters), Z-standardization of numerical variables, and fitting of a linear model to predict the outcomes. To improve the predictive performance through mitigation of overfitting, we performed L2 regularization of the model’s regression coefficients (Ridge regression), where the optimal regularization strength was determined in a nested 5-fold cross-validation loop in each training set.

Separately for both cohorts, we computed multivariable models that assess mental health at follow-up with all predictors combined. We used 10-fold cross-validation for a regularized linear and a nonlinear pipeline within the scikit-learn environment (Figures1  2 for details). To correct for chance effects, the analyses were repeated 10 times with different random number generator seeds, and the results were averaged (so that the validation altogether amounts to a 10 times repeated 10-fold cross-validation). Nonlinear regression can improve the accuracy of predictions by modeling nonlinear relationships between predictors and outcomes, for example, in the form of interactions (ie, the effect of social media consumption on future mental health might depend on gender) or nonlinear effects of single predictors (ie, very low or very high BMI values might both predict worse mental health). We used gradient boosting models (GBM [30]), which are currently considered the most accurate predictive tools for tabular datasets [31]. Specifically, we applied histogram-based GBMs as implemented in the HistGradientBoosting classes of scikit-learn for Python, which can automatically handle categorical and missing data. Hence, no imputation and encoding of categorical variables was performed as preprocessing in the nonlinear pipeline. The predictive performance was assessed with Pearson correlation coefficients and the mean squared error between predicted and true SDQ-E scores at follow-up in test set participants.

**Figure 2.**
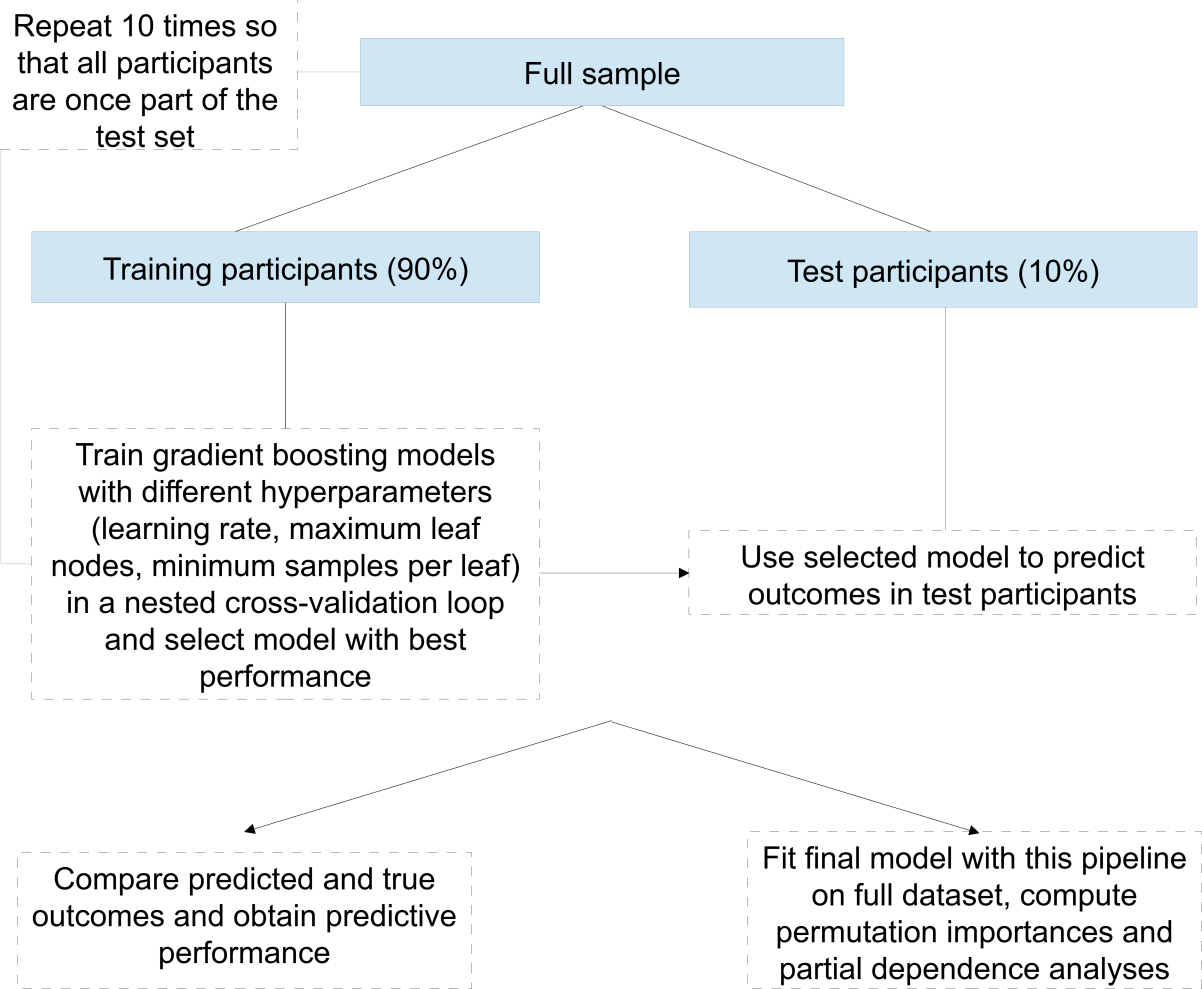
The nonlinear pipeline comprised fitting of a gradient boosting regression model to predict the outcomes, where the optimum value of crucial hyperparameters was determined in a nested 5-fold cross-validation loop in each training set. Iterations (adding of new trees to the predictor) were stopped when the predictor’s performance did not improve over the last 10 iterations. Due to the model’s native ability to handle missing values and categorical variables, no such preprocessing (imputation and encoding) was necessary.

### Predictor Importance Estimation

To obtain a measure of the importance of individual predictors in the multivariable models, we calculated permutation-based feature importances as implemented in the PermutationImportance class of the Alibi explain toolbox for Python [32]. This technique assesses the decline in a model’s performance when the associations between predictor and outcome are removed by randomly interchanging the values of 1 feature. The magnitude of decline in the model’s performance afforded by this permutation quantifies the extent to which the model relies on that specific predictor [33]. Here, we report the percentage change in performance when the predictor is intact compared to when it is perturbed.

Since the importance of individual predictors in multivariable models with many predictors is difficult to interpret (ie, the importance of a predictor decreases when a correlated predictor is added), we, moreover, applied a variant of this algorithm, which calculated the importance of a priori defined domains of predictors (Tables S1 and S2 in Multimedia Appendix 1) domains of predictors. The procedure here was identical to that for individual predictors (the predictors belonging to a domain are permuted together, and the associated decline in predictive performance is determined). To correct for potential chance effects, we fitted 10 GBMs with different random generators and averaged the predictor importances over these 10 models.

### Causal Effects of Predictor Changes

**Figure 3.**
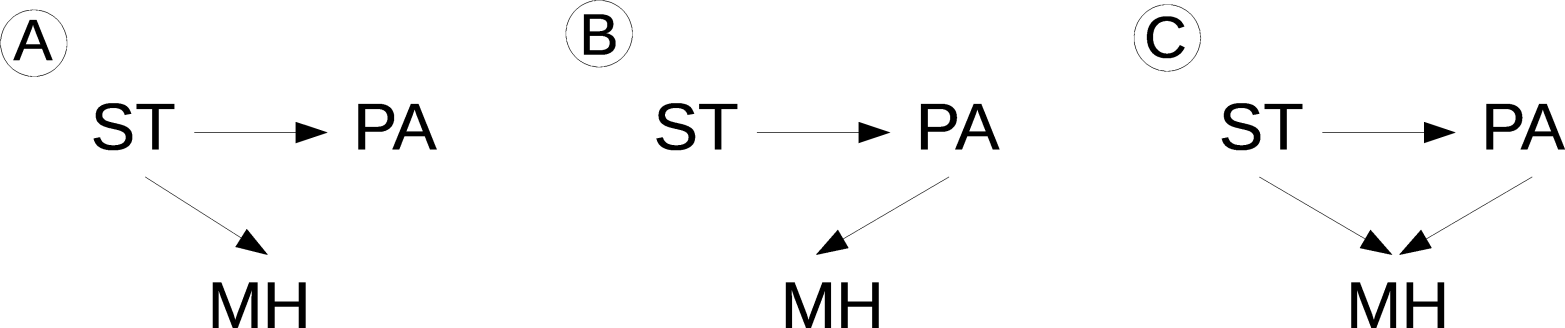
Exemplary possible causal relationships between screen time (ST), physical activity (PA), and mental health (MH). (**A**) Screen time could reduce physical activity and worsen mental health. Screen time thus generates a statistical relationship between physical activity and mental health, although a causal relationship is missing in this example. In this case, a partial dependence analysis would falsely show an improvement in mental health due to an increase in physical activity if screen time is not included in the model. The inclusion of screen time in the model closes this “backdoor path,” and conditional on screen time, physical activity and mental health become independent. A partial dependence analysis in this case (ie, with inclusion of screen time in the model) would correctly show an improvement in mental health through a reduction in screen time but not through an increase in physical activity. (**B**) High screen time could reduce physical activity and (only) thereby worsen mental health. In this case, without including physical activity in the model, there would be a statistical relationship between screen time and mental health. Conditioned on physical activity (by including this predictor in the model), screen time and mental health become statistically independent. If physical activity is not included in the model, a partial dependence analysis would show an improvement in mental health afforded by a reduction in screen time. However, if physical activity is included, a partial dependence analysis would show no effect of a reduction in screen time, but only of an increase in physical activity. (**C**) High screen time could have a direct negative effect on mental health and an additional indirect (mediated) effect by reducing physical activity. Without including physical activity in the model, in this case both the direct and the indirect (mediated by physical activity) effect on mental health would be “attributed” to screen time, and a partial dependence analysis would accordingly show the total effect (direct plus indirect effect) of a reduction in screen time. Including physical activity in the model would “attribute” the effect of screen time mediated by physical activity to physical activity. In this case, a partial dependence analysis would only show the direct effect of screen time on mental health.

We assessed the causal effect of factors whose change is discussed as the cause of the observed adolescent mental health crisis (physical inactivity, screen time, peer problems). In contrast to linear models, nonlinear regressors such as GBMs do not have simple coefficients that capture the adjusted influence of a predictor on the outcome. Hence, we artificially changed the baseline values of the relevant predictors for all participants to estimate the adjusted effects of these factors on mental health development during adolescence. All other predictors were kept unchanged, and we assessed mental health with these changed predictor values. This approach is similar to partial dependence plots [34] and can yield causal estimates when “backdoor paths” [35] between changed predictor and outcome are closed through appropriate inclusion of covariate predictors in the model [17]. However, ensuring that all backdoor paths are closed would require complete knowledge of the real causal graph, which is hardly possible in practice. Therefore, heuristics were developed for the best possible selection of predictor covariates to isolate causal effects, which can be applied with incomplete knowledge of the causal graph [18]. In line with this approach, we included available assessments of causes of mental health and of the factors investigated for causal effects on mental health into the predictive models. It should be noted that since this approach might lead to the inclusion of variables on the causal path from predictor to outcome (ie, effect mediators), the total causal effect (ie, the direct effect plus the effect indirectly mediated by mediators) of the predictors cannot be determined in this way [17,18]. Thus, the influence of the calculated predictors calculated is not an answer to the question, “How would mental health change in late adolescence if this predictor had a different value in early adolescence and the values of all other variables affected by this predictor also changed accordingly?” but rather to the question, “How would mental health change in late adolescence if this predictor had a different value in early adolescence but all other variables (including those potentially affected by the changed predictor) remained the same?” (Figure 3). This means that if an effect is found for a predictor in such an analysis, it represents a direct (ie, not conveyed via other mediators included in the model) cause of the further development of mental health.

Specifically, we tested the effect of screen time in both cohorts by setting all questions relating to time spent with media consumption to the minimum value (lowest), the 1/3 percentile (low), the 2/3 percentile (high), and the maximum value (highest). In both cohorts, these were questions about the hours spent using TV, computers, cell phones, and social media per day (details on all items in Tables S1 and S2 in Multimedia Appendix 1). Similarly, we set the values for questions on physical inactivity in both cohorts to minimum, 1/3 percentile, 2/3 percentile, and maximum and tested the impact on predicted mental health in late adolescence. These were questions on the number of days spent last week doing moderate to vigorous physical activity and the total minutes in moderate to vigorous physical activity as objectively recorded by the accelerometer in the MC and questions about the total hours of sports per week and the number of days physically active in the last 7 days in the KiGGS cohort (Tables S1 and S2 in Multimedia Appendix 1 for details). For peer problems, we changed parent-reported peer problems (as assessed in the SDQ with items like “The child has at least one good friend” or “The child is generally liked by other children”), the assessment of happiness with friendships, the feeling that “there is no one I feel close to,” and that “there is someone I trust whom I would turn to if I had problems” in the MC. Similarly, we changed the parent- and self-reported peer problems in the SDQ and the social support scale sum score value in the KiGGS cohort. Again, details on the items used to measure peer problems can be found in Tables S1 and S2 in Multimedia Appendix 1.

Similar to the feature importance estimation, we fitted 10 GBMs with different random generators and averaged the predicted mental health under all predictor changes over these 10 models to compensate for random calculations during model training.

### Data and Code Availability and Adherence to Reporting Guidelines

The analysis code used to generate the study results is freely available at the GitHub page of the authors[36]. For privacy reasons, the data cannot be made public, but data access can be requested at the institutes implementing the cohort studies (MC: Center for Longitudinal Studies of the University College London; KiGGS cohort: Department of Epidemiology and Health Monitoring of the Robert Koch Institute). The reporting of the model development and validation followed the Transparent Reporting of a multivariable prediction model for Individual Prognosis Or Diagnosis (TRIPOD [37]) guidelines (Table S4 in Multimedia Appendix 1).

## Results

### Univariable Relationships Between Baseline Predictors and Future Mental Health

**Figure 4.**
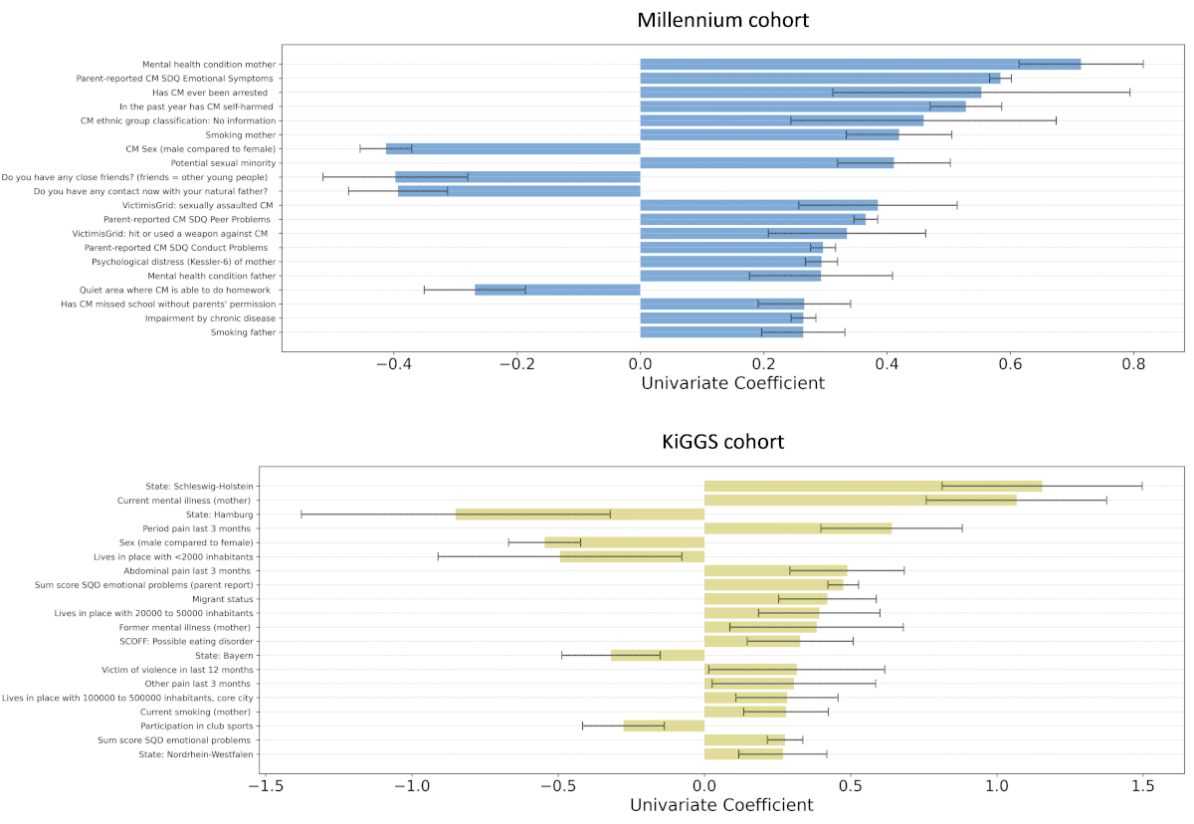
Univariable (unadjusted) linear relationships and 95% confidence intervals between predictors assessed at baseline and mental health (Strength and Difficulties Questionnaire—Emotional [SDQ-E]) at follow-up for the Millennium Cohort (upper row) and the KiGGS cohort (lower row). For reasons of conciseness, only the 20 most important predictors with statistical significance are shown and sorted in descending order of absolute coefficients. A complete overview of all predictors, including their univariable coefficients and their assignment to predictor domains, can be found in Tables S1 and S2 in Multimedia Appendix 1. For binary predictors, a “yes”-response was generally coded as 1, meaning that a negative value indicates less future mental health problems when the question was answered with “yes.” Ordinal-scaled items were ordered so that higher values indicated stronger agreement with a question.

The strongest statistically significant unadjusted linear relationships between baseline predictors and mental health at follow-up are shown in Figure 4 and coefficients of all predictors can be found in Tables S1 and S2 in Multimedia Appendix 1. Previous mental health problems, mental health problems of the parents, conduct problems, physical health problems and pain, low socioeconomic status, female sex, victimization, and potential exposure to marginalization and discrimination were related to worse future mental health in both cohorts.

### Predictive Performance of Multivariable Models

The average cross-validated predictive performance of the multivariable linear and nonlinear models is summarized in Table 2 (Pearson correlation coefficients between predicted and true SDQ-E scores as well as mean squared error). In both cohorts, the nonlinear model performed better on average and was hence used for further analyses.

**Table 2.** Predictive performance (linear correlation between predicted and true SDQ-E^a^ scores in test set participants) for different models and both cohorts (mean value and SD of 10 repeats of 10-fold cross-validation).

Model	Millennium cohort		KiGGS^b^ cohort	
	*r* (SD over cross-validation repeats)	MSE^c^ (SD over cross-validation repeats)	*r* (SD over cross-validation repeats)	MSE (SD over cross-validation repeats)
Linear	0.614 (.002)	3.203 (.005)	0.384 (.02)	2.869 (.02)
Nonlinear (gradient boosting)	0.614 (.002)	3.194 (.009)	0.466 (.03)	2.754 (.04)

a SDQ-E: Strength and Difficulties Questionnaire—Emotional.

b KiGGS: German Health Interview and Examination Survey for Children and Adolescents.

c MSE: mean squared error.

### Predictor Importance

**Figure 5.**
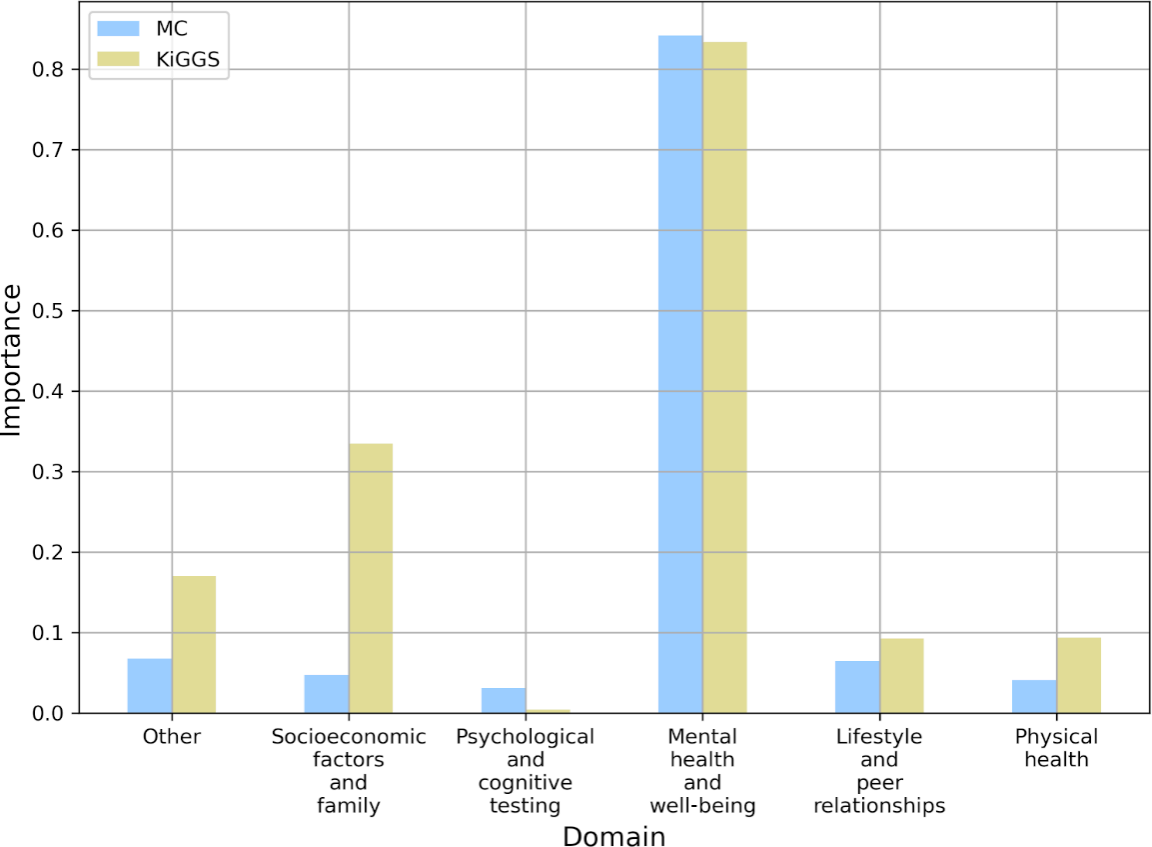
Permutation-based feature importance of predictor domains (percentage improvement by keeping the predictor domain intact compared to perturbing it).

The permutation-based importance of defined predictor domains is shown in Figure 5. Mental health assessments at baseline were the most important domain in both cohorts, but other predictors (participants sex, physical health such as chronic diseases, sociodemographic factors such as parental education and mental health, and lifestyle factors such as physical activity) also contributed to the predictions. The importance of each individual predictor as well as their assignments to predictor domains can be found in Tables S1 and S2 in Multimedia Appendix 1.

### Direct Effects of Screen Time, Physical Activity, and Peer Relationships

**Figure 6.**
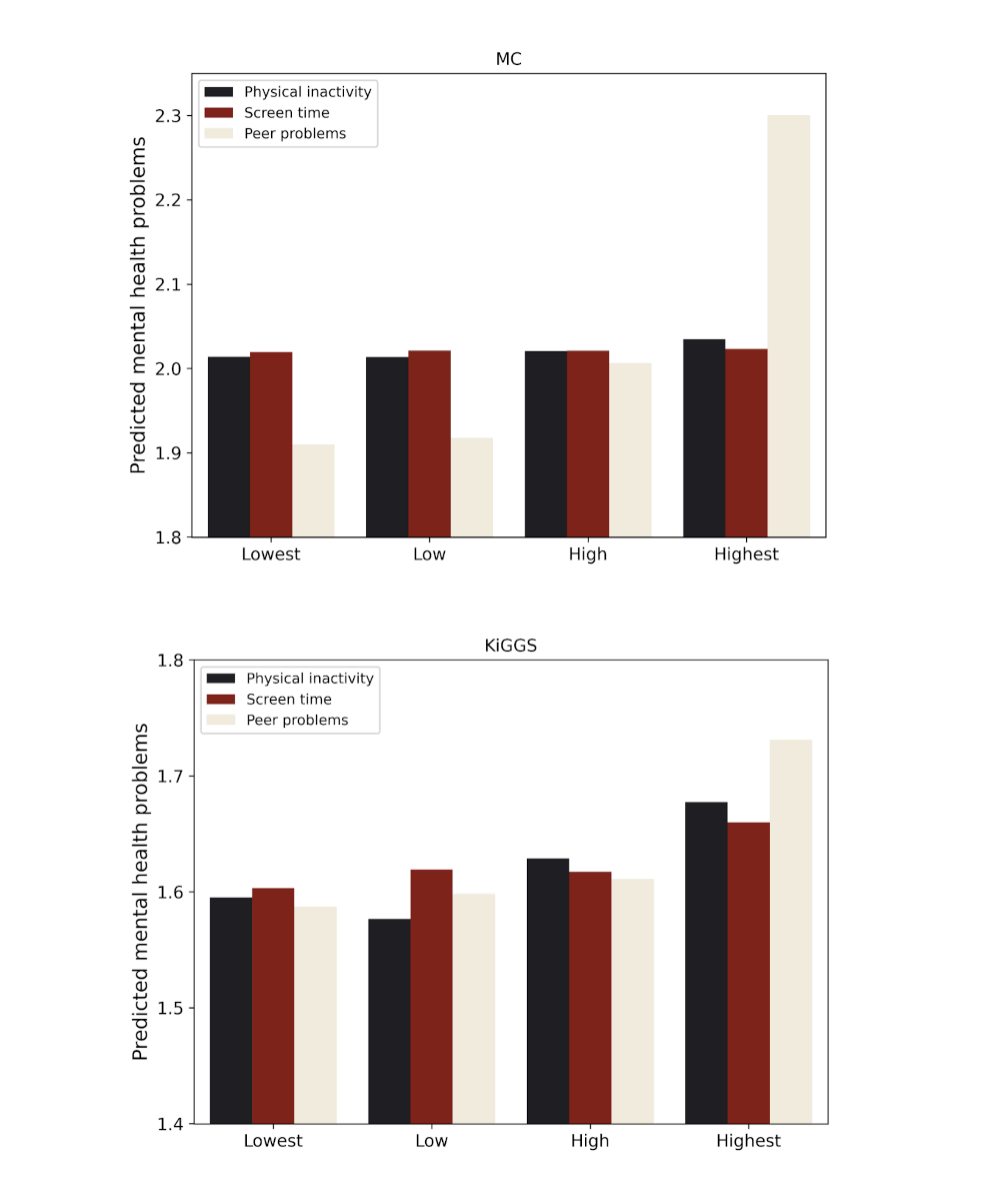
Direct effect of setting the value of predictors pertaining to screen time, physical activity, and peer relationships in early adolescence to minimum (lowest), 1/3 percentile (low), 2/3 percentile (high), and maximum (highest) on predicted mental health in late adolescence (keeping all other predictors constant). Screen time variables comprised all questions relating to time spent with media consumption (TV, computer, cell phone, and social media). Physical inactivity variables comprised days last week spent doing moderate to vigorous physical activity and total minutes in moderate to vigorous physical activity as objectively recorded by the accelerometer in the MC and total hours of sports per week and number of days physically active in the last 7 days in the KiGGS cohort. Peer problems variables comprised parent-reported peer problems (in the SDQ), the assessment of happiness with friendships, the feeling that “there is no one I feel close to,” and that “there is someone I trust whom I would turn to if I had problems” in the MC and parent- and self-reported peer problems and the social support scale sum score value in the KiGGS cohort. In Tables S1 and S2 in Multimedia Appendix 1, the corresponding variables are color-coded in accordance with the figure.

The direct causal effects of physical inactivity, screen time, and peer problems in early adolescence on mental health in late adolescence are summarized in Figure 6. It can be seen that in both cohorts, predicted mental health problems in late adolescence increased with growing peer problems. Both cohorts also showed more predicted mental health problems for high levels of physical inactivity, although this effect was small in the Millennium Cohort. Increased screen time did not change predictions in the MC, while in KiGGS, predictions with the highest screen time levels showed slightly more mental health problems compared to lower levels. These results show that, independent of changes in other predictors, a deterioration in peer relationships in particular leads to a worse development of mental health in both cohorts.

## Discussion

In this study, we used interpretable machine learning pipelines on 2 large European cohort studies for 2 related goals: First, we tested how well and based on which assessments the development of mental health during adolescence can be assessed. Second, we assessed the causal effects that high screen time, low physical activity, and worsened peer relationships had on an unfavorable development of mental health. Because the analyzed time period lies in the critical years after 2010, we thereby added evidence to the discussion on reasons for the mental health crisis unfolding during that time period.

Using cross-validation, we showed that multivariable models had medium to high predictive accuracy with Pearson correlations between predicted and actual mental health in late adolescence between 0.38 and 0.61. Although direct comparisons are complicated by differences in the study design and outcome measures, this predictive accuracy is in the upper range of the few studies published to date on the prediction of mental health problems in adolescents using comprehensive multivariable models [38,39]. Predictive performance was improved with nonlinear GBMs, which are known to perform well for medium-sized tabular datasets [31]. However, these figures also make it clear that the calculations of the models should be interpreted as assessments and not as absolutely accurate predictions. The relative superiority of the nonlinear model compared with the linear model suggests that at least some relationships between the investigated predictors and the development of mental health are not optimally modeled in a linear and additive fashion. This is also reflected, for example, in the disproportionately strong increase in predicted mental health problems with very severe peer problems.

We used univariable and multivariable feature importance analyses to identify predictive factors. Here, we were able to replicate and extend previously described risk and resilience factors. As expected and consistently demonstrated in previous studies [40,41], mental health problems in early adolescence were the strongest predictors of mental health problems in late adolescence. This result captures a certain stability of mental health problems during adolescence, but the contribution of other factors to the predictions shows that the development of adolescent mental health depends on multiple determinants that go beyond a mere “autoregressive” continuation of baseline mental health problems. Among family factors, mental health problems of the parents were strongly related to mental health problems in their adolescent children. For preventive strategies, it is important to understand the mechanisms of this intergenerational transmission of risk and to develop targeted interventions based on this understanding [42]. Indicators of physical health problems (chronic health conditions, self-reported pain, and impairments in general health) were also associated with future mental health problems. This finding substantiates current evidence that physical health problems in early life predict a worsening of mental health in adolescence and that children with chronic conditions might hence be particularly in need of support [23]. Low socioeconomic status (ie, income and education) of the parents predicted an unfavorable development of their children’s mental health during adolescence, which corroborates previous findings with different cohorts [43,44]. In both cohorts, we moreover found that female sex predicted worse mental health in late adolescence also when statistically correcting for baseline mental health symptoms in young adolescence. This finding is in accordance with a well-established gender gap in depressive symptoms, which starts to manifest during puberty, increases until adulthood, and has multifaceted reasons [28].

We leveraged recent advances in covariate selection and causal interpretation of predictive models [17,18] to approach the causal impacts of factors implicated in the deterioration of adolescent mental health in the past decade (less physical activity, more screen time, and worse peer relationships). This analysis revealed improved predicted mental health in late adolescence when more physical activity was assumed in early adolescence. However, the direct effect was small, particularly in the MC. We found that, except small effects of very much screen time in the KiGGS cohort, reducing screen time alone did not directly entail meaningful improvements in the predicted development of mental health in both cohorts. Conversely, good peer relationships predicted better mental health trajectories in both cohorts even when all other factors were held constant.

What do these results suggest in the context of current debates on the causes of the youth mental health crisis?

First, according to these analyses, adolescents would have had a more favorable mental health trajectory during the period of the observed deterioration in adolescent mental health if they had better peer relationships at ages 12-14 years, even if a wide range of other determinants of mental health had remained unchanged. It is noteworthy in this context that an overall deterioration in peer relationships of adolescents was observed across countries during the same period as the deterioration of mental health [45] and that proposed influencing factors such as increased screen time were shown to potentially have a detrimental impact on peer relationships [46]. It thus seems plausible that increased peer problems, themselves the result of a complex change in behavioral patterns of adolescents, represent an important direct cause of the deterioration of mental health. Given the growing evidence for the effectiveness of general prevention programs (such as antibullying school interventions) and individual therapeutic strategies (such as social skills training or self-esteem reflections) to improve peer relationships [47], it appears that a combination of these interventions would be a promising public health approach to the adolescent mental health crisis.

Second, adolescents would not have altered mental health trajectories if they had less screen time at ages 12-14 years when the other determinants of mental health had remained unchanged. This finding ties well with a recent panel network analysis showing that when multiple determinants of mental health were taken into account, social media use only had a very small influence on the development of mental health [48]. There is an intense debate around relationships between screen time, particularly social media use, and mental health in adolescents and young adults. The debate is fueled by a remarkable coincidence between the mass spread of social media and the deterioration of adolescent mental health around 2010 [49]. However, longitudinal studies could not prove a clear association between social media use and adolescent mental health [50,51]. It seems plausible that heavy social media use overall is part of a complex change in adolescent behaviors that might, potentially mediated through increased peer problems, contribute to mental health problems [11]. As illustrated in Figure 3, the absence of a direct effect in our study would be compatible with a model in which screen time exerts a negative effect on mental health via other downstream factors (eg, factors impacted by screen time and themselves impacting mental health, such as time spent with friends, body dissatisfaction, and sleep problems). Such modifiable factors on the causal pathway between exposures such as screen time and mental health outcomes could be targets for intervention strategies. Characterizing them more precisely would be an important undertaking for future research, which could use causal inference in combination with higher frequency surveys [18].

Third, adolescents would have a slightly improved mental health trajectory if they had been more physically active at ages 12-14 years, even when the other determinants of mental health had remained unchanged, especially those with very low levels of physical activity. In line with this finding, exercise interventions were shown to have small beneficial effects on adolescent mental health [52], and a recent review of reviews concluded that there is only “partial evidence” for a causal impact of physical activity on adolescent mental health [53]. Interestingly, the authors of this review discuss the possibility that the association between physical activity and adolescent mental health “may be linear, curvilinear, or contain a threshold, after which no further gains in mental health are made” [53], which would be consistent with our findings that increases in physical activity only have a beneficial direct effect in the case of low baseline levels. However, the small effect strengths, together with meta-studies that used aggregate data to demonstrate that the level of physical activity in adolescents has not relevantly decreased in Western countries between 2001 and 2016 [54], render it unlikely that the observed substantial worsening of adolescent mental in the past decade is due to decreased physical activity.

Some limitations of our study are relevant when interpreting the study results. First, causal inference from observational data is challenging, and results can depend on assumptions that are hard to test empirically. If the research questions cannot be investigated in a randomized controlled manner and one has to resort to observational data (as in our case), the best possible selection of covariates must be undertaken to approach causal interpretations. We applied principles developed for this purpose in our study [18]. Nevertheless, it should be kept in mind that the evidence for causal relationships in observational studies is generally lower compared to randomized controlled trials [55]. The results should therefore be interpreted in conjunction with other research work (eg, cohort studies, clinical observations, and analysis of aggregated time series) in order to gain a comprehensive picture of the factors contributing to the deterioration of young people’s mental health. Moreover, the inability to reliably distinguish confounders from mediators only allows for the calculation of direct effects, and it is possible that the factors under investigation have additional effects through the modification of mediator variables. To characterize such mediating relationships more precisely, future research could explore multiple-wave longitudinal studies in conjunction with causal inference [18]. Second, although the mutual validation of results in 2 independent cohorts is a strength, both cohorts differed in some design features, which reduces the comparability. In particular, the MC is age-homogenous, whilst in the age-heterogeneous KiGGS cohort, participants with an age similar to the MC participants were selected, leading to a smaller case number. Due to a longer interval between baseline and follow-up examination, participants in the KiGGS cohort were younger at baseline. This could contribute to observed discrepancies, for example, to the result that the direct effect of physical activity was more pronounced in the KiGGS cohort. In addition, predictors were only partially overlapping between the 2 studies. Despite these differences, it is noteworthy that key predictors of future mental health, such as the parents’ mental health, the participants’ baseline mental health, sex, peer problems, and physical activity, were found in both cohorts and there was a qualitative agreement on the estimated direct causal effects. The robustness of these results would be further strengthened by an analysis of additional cohorts from other countries. Third, not all proposed driving factors of the observed adolescent mental health deterioration could be investigated with our study. For instance, factors affecting society as a whole, such as growing social inequality and pressure to perform in education, have also been suggested [56,57] but could not be analyzed with our research design, which leveraged interindividual differences in risk factors to assess their impact on mental health development. Relatedly, the influence of new stressors such as the COVID pandemic, the war in Ukraine, and the increasingly noticeable effects of climate change could not be examined due to the time period under study. However, once appropriate data are available, our ML pipeline could be readily adapted in future work to predict the risk for mental health problems in the face of societal crises and identify its determining factors.

In summary, we showed that the development of adolescent mental health can be assessed by a combination of variables from early adolescence. Our analyses suggest a strong influence of peer relationships, and a deterioration of these through different reasons may contribute as a causal factor to the observed adolescent mental health crisis. Interventions to improve social relationships with peers could be explored as countermeasures against further deterioration of adolescent mental health.

## Supplementary material

10.2196/60125 Multimedia Appendix 1 Additional content.

## Abbreviations

GBM: gradient boosting model
KiGGS: German Health Interview and Examination Survey for Children and Adolescents
MC: British Millenium cohort
SDQ: Strength and Difficulties Questionnaire
SDQ-E: Strength and Difficulties Questionnaire—Emotional
TRIPOD: Transparent Reporting of a multivariable prediction model for Individual Prognosis Or Diagnosis
