# Supplementary material for: Peer Relationships Are a Direct Cause of the Adolescent Mental Health Crisis: Interpretable Machine Learning Analysis of 2 Large Cohort Studies
Source: JMIR Public Health Surveill. 2025 May 12;11:e60125. doi: 10.2196/60125 (PMC12088615; doi:10.2196/60125)
Supplement: Multimedia Appendix 1 [file publichealth-v11-e60125-s001.docx]

Peer relationships are a proximate cause of the adolescent mental health crisis: an interpretable machine learning analysis of two large cohort studies

Supplementary Content

[Supplementary Text 1. Testing the prespecified predictor domains 2](#_Toc163049183)

[Supplementary Text 2. Testing the quality of imputations 2](#_Toc163049184)

[Supplementary Figure 1. Participant flow chart for the Millennium and KiGGS cohort 3](#_Toc163049185)

[Supplementary Table 1. All predictors, univariate coefficients and multivariate feature importances of the Millennium Cohort 4](#_Toc163049186)

[Supplementary Table 2. All predictors, univariate coefficients and multivariate feature importances of the KiGGS Cohort 14](#_Toc163049187)

[Supplementary Table 3. Transparent reporting of a multivariable prediction model for individual prognosis or diagnosis (TRIPOD) checklist 22](#_Toc163049188)

# Supplementary Text 1. Testing the prespecified predictor domains

In order to simplify the interpretation of the feature importance analyses and to stabilize the results against the inclusion or exclusion of individual predictors, we investigated the permutation-based importance of predictor domains in addition to individual predictors. To this end, the predictors were assigned to the domains mental health and wellbeing, physical health, psychological and cognitive testing, socioeconomic and family factors, lifestyle and peer relationships, and other based on consultations with experts in adolescent mental health (URS & RS). In contrast to data-driven dimension reduction techniques, the focus here was not on an explanation of the highest possible variance of the predictors by the factors, but on their conceptual interpretability. However, with a meaningful assignment of predictors to domains, it should be expected that the predictors can be better explained by the domains assigned to them than by random assignments. To test this, we used the Python semopy package for structural equation modeling [1] and modeled the six domains as a linear combination of their assigned predictors (using the default semopy fit parameters and a small L2 regularization with regularization strength c = 1). Subsequently, we compared the fit (root mean squared error of approximation, RMSEA) of this model with 100 random models, in which the domain assignments were randomly permuted. The RMSEA of the model with our chosen domain assignments was 0.051 in the Millennium Cohort, here all 100 randomly perturbed assignments has RMSEA values > 0.053, and 0.050 in the KIGGS cohort, where all 100 randomly perturbed assignments had RMSEA values > 0.052. These results show that the domains and the predictors assigned to them, although based on purely content-related considerations, also statistically capture a latent factor structure of the data.

# Supplementary Text 2. Testing the quality of imputations

In our study, the imputation algorithm was trained in the training set and applied in the test set of the cross-validation folds. The exact algorithm depended on the analysis pipeline: KNN imputation with default parameters of the KNNImputer class of scikit-learn was used for the univariable analyses and the linear multivariable analyses. No other non-default parameters were evaluated. For the nonlinear multivariable analyses, we relied on the native treatment of missing values in the HistGradientBoostingRegressor implementation. Here, during training, the tree grower learns at each split point whether samples with missing values should go to the “left” or “right” side of the split, based on the potential loss. When predicting, samples with missing values are assigned to the “left” or “right” leaf node accordingly.

To exploratively obtain a metric on the quality of values imputed with the KNNImputer, we randomly set 5% of the predictor values to missing, imputed them with this method and calculated Pearson correlation coefficients between imputed and true values. This analysis yielded a correlation of r = 0.448, p < 0.001 for the Millennium Cohort and r = 0.300, p < 0.001 for the KIGGS cohort. Based on these analyses, it can be assumed that the quality of the imputed values with KNN imputations was better than with frequently used univariate imputation methods such as mean or median imputation.

# Supplementary Figure 1. Participant flow chart for the Millennium and KiGGS cohort

To be included in the analyses, participants had to:

a) be 11 years or older at baseline

b) have a valid SDQ-E assessment (analysis outcome) at follow-up

c) have less than 80% of missing predictors at baseline

As shown in the participant flow chart below, criteria (a) and (b) led to the exclusion of many KIGGS participants. The reason for this is that the KIGGS cohort was age-heterogeneous, i.e. many participants were 10 years or younger at baseline or already adults at follow-up, which is why the SDQ, which is only validated for minors, was no longer used. By excluding these participants, an approximately age-homogeneous structure was achieved that was similar to that of the Millennium Cohort.

**
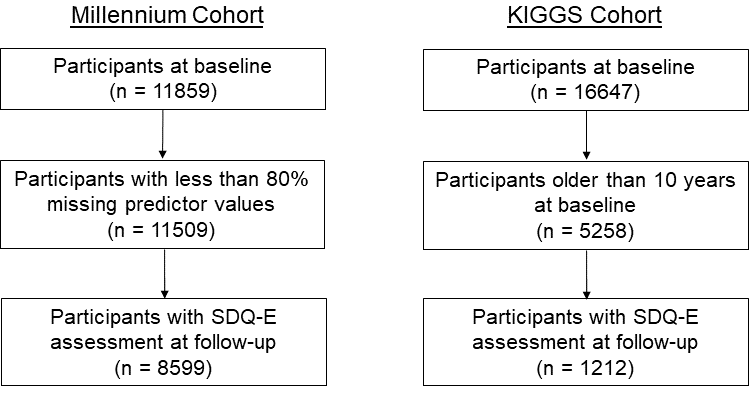
**

For comparison with KIGGS-related publications, it should be noted that our baseline sample is a selection of participants from KIGGS Wave 1 and our follow-up sample is a selection of participants from Wave 2.

# Supplementary Table 1. All predictors, univariate coefficients and multivariate feature importances of the Millennium Cohort

A detailed description of the instruments including the original questionnaire is available at

https://cls.ucl.ac.uk/cls-studies/millennium-cohort-study/mcs-age-14-sweep/

▪ Variable is part of the physical inactivity assessment

▪ Variable is part of the screen time assessment

▪ Variable is part of the peer problems assessment

| **Variable Code** | **Variable Description** | **Domain assignment** | **Percent missing values** | **Univariate Association** | **Univariate Association CI** | **Multivariate Feature Importance** |
| --- | --- | --- | --- | --- | --- | --- |
| Area_Deprivation | Deprivation index of living area | Socioeconomic factors and family | 0 | 0.1229 | (0.1011, 0.1448) | 0.0001 |
| ChronicDisease | Impairment by chronic disease | Physical health | 0.67 | 0.2645 | (0.2443, 0.2847) | 0.0259 |
| FCALCD00 | Has CM ever had an alcoholic drink | Lifestyle and peer relationships | 1.52 | 0.0078 | (-0.0353, 0.0509) | 0.0001 |
| FCALFV00 | Has CM ever had 5 or more alcoholic drinks at a time | Lifestyle and peer relationships | 57.46 | 0.0666 | (-0.0064, 0.1395) | 0.0002 |
| FCARES00 | Has CM ever been arrested | Lifestyle and peer relationships | 1.38 | 0.553 | (0.3119, 0.7942) | 0 |
| FCATQL00 | Needs qualifications to get a job worth having | Psychological and cognitive testing | 6.07 | -0.0433 | (-0.0651,  -0.0216) | 0 |
| FCBAND00 | ActivGrid: Sing in a choir or play in a band or orchestra? | Lifestyle and peer relationships | 0.34 | -0.0034 | (-0.0255, 0.0187) | 0 |
| FCBGFR00 | Does CM have a boyfriend/girlfriend | Lifestyle and peer relationships | 1.6 | 0.0545 | (-0.0014, 0.1105) | 0.0001 |
| FCBMIN6 | MCS6 Body Mass Index calculated (CLS) | Physical health | 3.44 | 0.1051 | (0.0835, 0.1267) | 0.003 |
| FCBTNG00 | ConsumAttGrid: It bothers me if my friends have things I don't | Lifestyle and peer relationships | 0.41 | 0.0345 | (0.0133, 0.0556) | 0 |
| FCBULB00 | How often brothers or sisters hurt or pick on CM | Lifestyle and peer relationships | 1.5 | 0.0569 | (0.0352, 0.0787) | 0.0008 |
| FCBULP00 | How often CM hurts or picks on brothers or sisters | Lifestyle and peer relationships | 7.81 | 0.0382 | (0.016, 0.0604) | 0.0004 |
| FCCANB00 | DruGrid: Cannabis (also known as weed, marijuana, dope, hash or skunk)? | Lifestyle and peer relationships | 1.49 | 0.1036 | (-0.004, 0.2112) | 0.0002 |
| FCCDDL00 | (last 12 months) has CM cuddled with another young person? | Lifestyle and peer relationships | 2.05 | -0.0996 | (-0.1428,  -0.0563) | 0.0004 |
| FCCGHE00 | CM's general level of health | Physical health | 0.36 | -0.2009 | (-0.2219,  -0.1799) | 0.0111 |
| FCCINE00 | ActivGrid: Go to the cinema? | Lifestyle and peer relationships | 0.13 | -0.091 | (-0.1124,  -0.0695) | 0.0028 |
| FCCMEX00 | Does CM have computer of their own | Lifestyle and peer relationships | 0 | -0.0519 | (-0.112, 0.0083) | 0 |
| FCCOFA00 | Do you have any contact now with your natural father? | Socioeconomic factors and family | 75.61 | -0.3931 | (-0.4733,  -0.3128) | 0 |
| ▪ FCCOMH00 | Hours per weekday spent playing electronic games | Lifestyle and peer relationships | 0.07 | -0.035 | (-0.0562,  -0.0139) | 0.0004 |
| FCCOPY00 | Wrong to download music/films/games without paying | Psychological and cognitive testing | 0.33 | 0.052 | (0.0306, 0.0733) | 0.0001 |
| FCCSEX00 | CM Sex | Other | 0 | -0.4124 | (-0.4543,  -0.3706) | 0.0628 |
| FCCYBO00 | How often CM bullied other children online | Lifestyle and peer relationships | 1.56 | 0.0485 | (0.0273, 0.0696) | 0 |
| FCCYBU00 | How often other children bullied CM online | Lifestyle and peer relationships | 1.58 | 0.143 | (0.1209, 0.165) | 0.0004 |
| FCDISG00 | DisciplineGrid: Ground you, stop you going out or from seeing your friends? | Socioeconomic factors and family | 3.34 | 0.0832 | (0.0392, 0.1272) | 0.0003 |
| FCDISP00 | DisciplineGrid: Punish you in some other way? | Socioeconomic factors and family | 4.36 | 0.0284 | (-0.0188, 0.0755) | 0.0001 |
| FCDIST00 | DisciplineGrid: Tell you off or shout at you? | Socioeconomic factors and family | 2.17 | 0.08 | (0.0029, 0.1572) | 0.0002 |
| FCDOWL00 | RosenbergGrid: I am able to do things as well as most other people | Mental health and wellbeing | 1.83 | -0.2157 | (-0.2365,  -0.1948) | 0.0005 |
| FCETLS00 | Has CM ever eaten less to lose weight | Mental health and wellbeing | 1.81 | 0.2105 | (0.1673, 0.2537) | 0.0001 |
| FCEXWT00 | Has CM ever exercised to lose weight | Mental health and wellbeing | 1.66 | 0.0705 | (0.0266, 0.1143) | 0 |
| FCFGHT00 | Wrong to start a fight | Psychological and cognitive testing | 0.24 | 0.0216 | (0.0001, 0.0431) | 0 |
| FCFMLY00 | WellbeingGrid: How happy is CM with family | Mental health and wellbeing | 1.69 | -0.1841 | (-0.2053,  -0.1628) | 0.0004 |
| ▪ FCFRNS00 | WellbeingGrid: How happy is CM with friends | Mental health and wellbeing | 1.63 | -0.1902 | (-0.2111,  -0.1692) | 0.0028 |
| FCGANG00 | Is CM a member of a street gang | Lifestyle and peer relationships | 1.48 | 0.2287 | (0.0894, 0.368) | 0 |
| FCGDQL00 | RosenbergGrid: I feel I have a number of good qualities | Mental health and wellbeing | 1.83 | -0.1974 | (-0.2185,  -0.1763) | 0.0006 |
| FCGDSF00 | RosenbergGrid: I feel good about myself | Mental health and wellbeing | 1.9 | -0.2251 | (-0.2458,  -0.2043) | 0.0004 |
| FCGTDELAY | CGT Delay Aversion | Psychological and cognitive testing | 5.9 | 0.0437 | (0.0215, 0.0659) | 0.0052 |
| FCGTDTIME | CGT Deliberation Time | Psychological and cognitive testing | 5.87 | 0.099 | (0.078, 0.1199) | 0.003 |
| FCGTQOFDM | CGT Quality of Decision Making | Psychological and cognitive testing | 5.87 | -0.0716 | (-0.0936,  -0.0495) | 0.0013 |
| FCGTRISKA | CGT Risk adjustment | Psychological and cognitive testing | 5.87 | -0.1251 | (-0.1467,  -0.1035) | 0.0028 |
| FCGTRISKT | CGT Risk taking | Psychological and cognitive testing | 5.87 | -0.0746 | (-0.0966,  -0.0526) | 0.0029 |
| FCHARM00 | In the past year has CM self-harmed | Mental health and wellbeing | 1.8 | 0.528 | (0.4701, 0.5859) | 0.0009 |
| FCHHND00 | (last 12 months) has CM held hands with another young person? | Lifestyle and peer relationships | 2.08 | -0.1462 | (-0.1898,  -0.1026) | 0.0015 |
| FCHITT00 | ASBOgrid: CM Pushed or shoved/hit/slapped/punched someone? | Lifestyle and peer relationships | 1.5 | -0.0085 | (-0.0557, 0.0387) | 0 |
| FCHLPC00 | How often does anyone at home help with CM's homework | Socioeconomic factors and family | 0.19 | -0.0829 | (-0.1044,  -0.0615) | 0 |
| FCHURT00 | How often other children hurt or pick on CM | Lifestyle and peer relationships | 1.59 | 0.1739 | (0.153, 0.1947) | 0.0017 |
| FCHWKM00 | typical week term-time, how long cm spends doing homework? | Lifestyle and peer relationships | 0.2 | -0.0805 | (-0.1016,  -0.0594) | 0.0002 |
| FCIMWK00 | GenderRolesGrid: It is less important for women to work than it is for men | Other | 1.12 | 0.0282 | (0.0066, 0.0497) | 0.0049 |
| FCINTH00 | Does CM ever use the internet at home | Lifestyle and peer relationships | 0.03 | 0.055 | (0.0339, 0.0762) | 0.0001 |
| FCKISS00 | (last 12 months) has CM kissed another young person? | Lifestyle and peer relationships | 1.93 | -0.0614 | (-0.106,  -0.0168) | 0 |
| FCLIFE00 | WellbeingGrid: How happy is CM with life as a whole | Mental health and wellbeing | 1.76 | -0.2506 | (-0.2715,  -0.2298) | 0.0031 |
| FCMDSA00 | FeelingsGrid: I felt miserable or unhappy | Mental health and wellbeing | 1.79 | 0.2326 | (0.2118, 0.2535) | 0.0009 |
| FCMDSB00 | FeelingsGrid: I didn't enjoy anything at all | Mental health and wellbeing | 1.86 | 0.1866 | (0.1657, 0.2075) | 0.0001 |
| FCMDSC00 | FeelingsGrid: I felt so tired I just sat around and did nothing | Mental health and wellbeing | 1.88 | 0.1604 | (0.1392, 0.1816) | 0.0002 |
| FCMDSD00 | FeelingsGrid: I was very restless | Mental health and wellbeing | 1.93 | 0.1797 | (0.1585, 0.201) | 0 |
| FCMDSE00 | FeelingsGrid: I felt I was no good any more | Mental health and wellbeing | 1.99 | 0.241 | (0.2204, 0.2615) | 0.0024 |
| FCMDSF00 | FeelingsGrid: I cried a lot | Mental health and wellbeing | 1.88 | 0.2438 | (0.2229, 0.2647) | 0.0007 |
| FCMDSG00 | FeelingsGrid: I found it hard to think properly or concentrate | Mental health and wellbeing | 1.91 | 0.2062 | (0.1852, 0.2273) | 0.0009 |
| FCMDSH00 | FeelingsGrid: I hated myself | Mental health and wellbeing | 1.78 | 0.2464 | (0.2258, 0.2669) | 0.0061 |
| FCMDSI00 | FeelingsGrid: I was a bad person | Mental health and wellbeing | 1.83 | 0.1588 | (0.1378, 0.1798) | 0 |
| FCMDSJ00 | FeelingsGrid: I felt lonely | Mental health and wellbeing | 1.81 | 0.2388 | (0.2178, 0.2598) | 0.0004 |
| FCMDSK00 | FeelingsGrid: I thought nobody really loved me | Mental health and wellbeing | 1.84 | 0.1996 | (0.1787, 0.2205) | 0.0006 |
| FCMDSL00 | FeelingsGrid: I thought I could never be as good as other kids | Mental health and wellbeing | 1.81 | 0.2364 | (0.2156, 0.2572) | 0.0007 |
| FCMDSM00 | FeelingsGrid: I did everything wrong | Mental health and wellbeing | 1.79 | 0.2164 | (0.1957, 0.2371) | 0.0001 |
| FCMISB00 | How often does CM misbehave in lessons | Lifestyle and peer relationships | 0.24 | 0.015 | (-0.0064, 0.0364) | 0.0002 |
| FCMISO00 | How often do other pupils misbehave in lessons | Socioeconomic factors and family | 0.24 | 0.1614 | (0.1401, 0.1826) | 0.0077 |
| FCMNWO00 | EducMotGrid: How often difficult to keep mind on work at school? [NCDS, BCS] | Mental health and wellbeing | 0.3 | 0.1755 | (0.1542, 0.1968) | 0.0005 |
| FCMUSM00 | ActivGrid: Go to museums/galleries, visit historic place/stately home? | Lifestyle and peer relationships | 0.13 | -0.053 | (-0.0743,  -0.0316) | 0.0001 |
| ▪ FCNCLS00 | SocSupGrid: There is no one I feel close to. | Lifestyle and peer relationships | 1.86 | 0.1189 | (0.0967, 0.141) | 0.0005 |
| FCNUFR00 | Do you have any close friends? (friends = other young people) | Lifestyle and peer relationships | 0.26 | -0.3975 | (-0.5149,  -0.2801) | 0 |
| FCONDUCT | Parent-reported CM SDQ Conduct Problems | Mental health and wellbeing | 2.56 | 0.2963 | (0.276, 0.3165) | 0.0113 |
| FCORGA00 | ActivGrid: Go to youth clubs/scouts/girlguides or oth organised activities? | Lifestyle and peer relationships | 0.13 | -0.082 | (-0.1033,  -0.0607) | 0.0001 |
| FCOTDR00 | DruGrid: Any other illegal drug (such as ecstasy, cocaine, speed)? | Lifestyle and peer relationships | 1.49 | 0.234 | (-0.0561, 0.5241) | 0.0001 |
| FCOTWD00 | When CM goes out, how often do parents know what CM does? | Socioeconomic factors and family | 1.49 | -0.0279 | (-0.0493,  -0.0065) | 0 |
| FCOTWI00 | When CM goes out, how often do parents know who with? | Socioeconomic factors and family | 1.58 | 0.0064 | (-0.0149, 0.0277) | 0.0001 |
| FCOUTW00 | When CM goes out, how often do parents know where? | Socioeconomic factors and family | 1.45 | -0.0394 | (-0.0609,  -0.0179) | 0 |
| FCPCKP00 | How often CM hurts or picks on other children | Lifestyle and peer relationships | 1.52 | 0.0231 | (0.0014, 0.0448) | 0 |
| ▪ FCPHEX00 | Days last week spent doing moderate to vigorous physical activity | Lifestyle and peer relationships | 0.14 | -0.1583 | (-0.1795,  -0.1371) | 0.0008 |
| FCPLAB00 | ConsumAttGrid: I like clothing with popular labels | Lifestyle and peer relationships | 0.35 | -0.056 | (-0.0774,  -0.0345) | 0.0001 |
| FCPLWE00 | Do you allow unsupervised time outside home with frds (on weekends) | Lifestyle and peer relationships | 0.06 | -0.0843 | (-0.1063,  -0.0624) | 0 |
| FCPLWK00 | Do you allow unsupervised time outside home with frds (afternoon after school) | Lifestyle and peer relationships | 0.07 | -0.0402 | (-0.0615,  -0.0188) | 0.0002 |
| FCPTNT00 | How patient is CM | Psychological and cognitive testing | 0.78 | -0.1266 | (-0.1482,  -0.105) | 0.0003 |
| FCQTRM00 | Quiet area where CM is able to do homework | Socioeconomic factors and family | 0.23 | -0.2685 | (-0.3507,  -0.1864) | 0 |
| FCQUAF00 | How often does CM argue with father | Socioeconomic factors and family | 7.59 | 0.0412 | (0.0191, 0.0632) | 0.0037 |
| FCQUAM00 | How often does CM argue with mother | Socioeconomic factors and family | 2.36 | 0.1297 | (0.1082, 0.1513) | 0.0025 |
| FCRISK00 | How willing is CM to take risks | Psychological and cognitive testing | 1.06 | -0.1438 | (-0.1653,  -0.1223) | 0.0061 |
| FCRJOY00 | ActivGrid: Read for enjoyment (not for school)? | Lifestyle and peer relationships | 0.29 | 0.0181 | (-0.0032, 0.0394) | 0.0057 |
| FCRLQF00 | How close is CM with father | Socioeconomic factors and family | 1.48 | -0.0796 | (-0.1012,  -0.058) | 0.0017 |
| FCRLQM00 | How close is CM with mother | Socioeconomic factors and family | 1.35 | -0.0769 | (-0.0982,  -0.0555) | 0.0009 |
| FCRLSV00 | ActivGrid: Attend a religious service? | Lifestyle and peer relationships | 0.17 | -0.0476 | (-0.0715,  -0.0237) | 0.0001 |
| FCROLE00 | GenderRolesGrid: Men and women should do the same jobs around the house | Other | 0.28 | 0.015 | (-0.0061, 0.0361) | 0 |
| FCSAFF00 | SocSupGrid: I have family and friends who help me feel safe, secure and happy. | Lifestyle and peer relationships | 1.48 | -0.1092 | (-0.1304,  -0.088) | 0 |
| FCSATI00 | RosenbergGrid: On the whole, I am satisfied with myself | Mental health and wellbeing | 1.93 | -0.2372 | (-0.2579,  -0.2165) | 0 |
| FCSCBE00 | EducMotGrid: How often do you try your best at school? | Mental health and wellbeing | 0.2 | -0.015 | (-0.0364, 0.0063) | 0 |
| FCSCHL00 | WellbeingGrid: How happy is CM with school | Mental health and wellbeing | 1.65 | -0.2047 | (-0.2259,  -0.1834) | 0.0016 |
| FCSCWA00 | EducMotGrid: How often do you feel school is a waste of time? | Mental health and wellbeing | 0.24 | 0.1057 | (0.0844, 0.1269) | 0.0001 |
| FCSCWK00 | WellbeingGrid: How happy is CM with school work | Mental health and wellbeing | 1.55 | -0.2061 | (-0.2272,  -0.1849) | 0.0033 |
| FCSINT00 | EducMotGrid: How often do you find school interesting? | Mental health and wellbeing | 0.19 | -0.0838 | (-0.1052,  -0.0625) | 0 |
| FCSLLN00 | During the last 4 weeks how long does going to sleep usually take | Mental health and wellbeing | 0.91 | 0.1235 | (0.1022, 0.1449) | 0.0003 |
| FCSLTR00 | During the last 4 weeks how often did CM awaken during sleep | Mental health and wellbeing | 0.58 | 0.1907 | (0.1696, 0.2118) | 0.0017 |
| FCSMOK00 | How often CM smokes cigarettes | Lifestyle and peer relationships | 1.87 | 0.0771 | (0.0564, 0.0978) | 0 |
| ▪ FCSOME00 | Hours per week spent on social networking sites | Lifestyle and peer relationships | 0.03 | 0.0286 | (0.0075, 0.0498) | 0 |
| FCSPNT00 | Wrong to write or spray paint on building/fence etc | Psychological and cognitive testing | 0.17 | -0.0343 | (-0.0556,  -0.0129) | 0.0001 |
| FCSPOR00 | ActivGrid: Go to watch live sport? | Lifestyle and peer relationships | 0.33 | -0.1657 | (-0.1867,  -0.1448) | 0.0057 |
| FCSTEL00 | Wrong to take things without paying | Psychological and cognitive testing | 0.17 | 0.0059 | (-0.015, 0.0269) | 0.0002 |
| FCSTFR0A | When not at school, how often do you spend time with your close friends? | Lifestyle and peer relationships | 3.38 | -0.0848 | (-0.1068,  -0.0629) | 0 |
| FCSTIR00 | EducMotGrid: How often do you get tired at school? | Mental health and wellbeing | 0.2 | 0.1637 | (0.1425, 0.1849) | 0.0007 |
| FCSTLN00 | ASBOgrid: CM stolen something from someone eg mobile phone, money | Lifestyle and peer relationships | 1.48 | 0.1812 | (-0.0159, 0.3782) | 0 |
| FCSTYU00 | How likely is it CM will go to university? (Scale 0-100%) | Psychological and cognitive testing | 4.24 | -0.1319 | (-0.1528,  -0.1111) | 0.0062 |
| FCSTYY00 | How likely is it CM will remain in education at the end of year 11? | Psychological and cognitive testing | 1.13 | -0.1341 | (-0.1556,  -0.1126) | 0.0035 |
| FCSUNH00 | EducMotGrid: How often do you feel unhappy at school? | Mental health and wellbeing | 0.26 | 0.2469 | (0.2261, 0.2677) | 0.0004 |
| ▪ FCTRSS00 | SocSupGrid: There is someone I trust whom I would turn to if I had problems | Lifestyle and peer relationships | 1.6 | -0.0835 | (-0.105, -0.0619) | 0.0001 |
| FCTRST0A | How much does CM trust others | Lifestyle and peer relationships | 0.63 | -0.2133 | (-0.2342,  -0.1923) | 0.0067 |
| FCTRUA00 | Has CM missed school without parents' permission | Lifestyle and peer relationships | 0.3 | 0.2659 | (0.1908, 0.341) | 0 |
| ▪ FCTVHO00 | Hours per weekday spent watching TV or videos on computer | Lifestyle and peer relationships | 0.07 | 0.0517 | (0.0307, 0.0727) | 0.0005 |
| FCVALU00 | RosenbergGrid: I am a person of value | Mental health and wellbeing | 2.57 | -0.1834 | (-0.2046,  -0.1622) | 0.0001 |
| FCVEGI00 | How often CM eats at least 2 portions of vegetables | Lifestyle and peer relationships | 0.47 | -0.1115 | (-0.1329,  -0.0901) | 0 |
| FCVICA00 | VictimisGrid: been physically violent towards CM | Lifestyle and peer relationships | 1.58 | 0.0864 | (0.0346, 0.1382) | 0 |
| FCVICC00 | VictimisGrid: hit or used a weapon against CM | Lifestyle and peer relationships | 1.6 | 0.3351 | (0.2074, 0.4627) | 0 |
| FCVICE00 | VictimisGrid: stolen something from CM | Lifestyle and peer relationships | 1.6 | 0.1936 | (0.1109, 0.2762) | 0.0001 |
| FCVICF0A | VictimisGrid: sexually assaulted CM | Lifestyle and peer relationships | 1.62 | 0.3853 | (0.2569, 0.5137) | 0 |
| FCVICG00 | VictimisGrid: CM insulted, threatened, shouted at | Lifestyle and peer relationships | 1.57 | 0.1995 | (0.1563, 0.2426) | 0 |
| FCWEGT00 | CM's perception of their weight | Mental health and wellbeing | 1.76 | 0.1214 | (0.0998, 0.1429) | 0.0001 |
| FCWELK00 | MoralAttGrid: How important is it to you to be well liked? | Lifestyle and peer relationships | 0.22 | -0.0584 | (-0.0795,  -0.0372) | 0.005 |
| FCWEPN00 | ASBOgrid: CM Used or hit someone with a weapon? | Lifestyle and peer relationships | 1.48 | 0.0492 | (-0.1943, 0.2927) | -0.0001 |
| FCWHRD00 | MoralAttGrid: How important is it to you to work hard? | Lifestyle and peer relationships | 0.22 | -0.0488 | (-0.0699, -0.0276) | 0 |
| FCWRDSC | CM Word activity score out of 20 | Psychological and cognitive testing | 5.28 | -0.0898 | (-0.1111,  -0.0684) | 0.0004 |
| FCWYLK00 | WellbeingGrid: How happy is CM with the way they look | Mental health and wellbeing | 1.69 | -0.2259 | (-0.2469,  -0.205) | 0.001 |
| FDAUDITf | AUDIT score father | Socioeconomic factors and family | 38.32 | -0.0584 | (-0.0834,  -0.0334) | 0.0007 |
| FDAUDITm | AUDIT score mother | Socioeconomic factors and family | 41.2 | -0.0622 | (-0.0888,  -0.0356) | 0.0003 |
| FDCE0600 | CM ethnic group classification | Socioeconomic factors and family | 0.85 |  |  | 0.002 |
| FDCE0600_0.0 | CM ethnic group classification: White | Socioeconomic factors and family |  | 0.0058 | (-0.0543, 0.0659) |  |
| FDCE0600_1.0 | CM ethnic group classification: Mixed | Socioeconomic factors and family |  | -0.0888 | (-0.1891, 0.0114) |  |
| FDCE0600_2.0 | CM ethnic group classification: Indian | Socioeconomic factors and family |  | -0.2121 | (-0.3686,  -0.0556) |  |
| FDCE0600_3.0 | CM ethnic group classification: Pakistani or Bangladeshi | Socioeconomic factors and family |  | 0.0167 | (-0.1008, 0.1341) |  |
| FDCE0600_4.0 | CM ethnic group classification: Black | Socioeconomic factors and family |  | -0.0638 | (-0.2105, 0.0829) |  |
| FDCE0600_5.0 | CM ethnic group classification: Other | Socioeconomic factors and family |  | 0.2267 | (0.0556, 0.3978) |  |
| FDCE0600_nan | CM ethnic group classification: No information | Socioeconomic factors and family |  | 0.4593 | (0.2441, 0.6744) |  |
| FDKESSLf | Psychological distress (Kessler-6) of father | Socioeconomic factors and family | 41.49 | 0.1262 | (0.101, 0.1514) | 0.0011 |
| FDKESSLm | Psychological distress (Kessler-6) of mother | Socioeconomic factors and family | 44.24 | 0.2938 | (0.2676, 0.3199) | 0.0065 |
| FDNEUROTf | Big five neuroticism of father | Socioeconomic factors and family | 40.89 | 0.1101 | (0.0839, 0.1363) | 0.0009 |
| FDNEUROTm | Big five neuroticism of mother | Socioeconomic factors and family | 43.34 | 0.214 | (0.1878, 0.2401) | 0.0057 |
| FDOTHS00 | Number of siblings of CM in household | Socioeconomic factors and family | 0 | 0.0054 | (-0.017, 0.0279) | 0.0005 |
| FEMOTION | Parent-reported CM SDQ Emotional Symptoms | Mental health and wellbeing | 2.55 | 0.5839 | (0.5662, 0.6016) | 0.6639 |
| FHYPER | Parent-reported CM SDQ Hyperactivity/Inattention | Mental health and wellbeing | 2.58 | 0.262 | (0.2417, 0.2823) | 0.0085 |
| FOEDE000 | OECD equiv weekly family income | Socioeconomic factors and family | 0.07 | -0.1741 | (-0.1948,  -0.1535) | 0.0039 |
| ▪ FPEER | Parent-reported CM SDQ Peer Problems | Lifestyle and peer relationships | 2.54 | 0.3655 | (0.3461, 0.3849) | 0.0245 |
| FPLOLR0Gf | Mental health condition father | Socioeconomic factors and family | 87.29 | 0.2928 | (0.1766, 0.409) | 0.0001 |
| FPLOLR0Gm | Mental health condition mother | Socioeconomic factors and family | 88.08 | 0.7148 | (0.6139, 0.8156) | 0.0013 |
| FPROSOC | Parent-reported CM SDQ Prosocial | Mental health and wellbeing | 2.54 | -0.1576 | (-0.1789,  -0.1363) | 0.0003 |
| FPSMUS0Af | Smoking father | Socioeconomic factors and family | 38.32 | 0.2641 | (0.1964, 0.3319) | 0.0002 |
| FPSMUS0Am | Smoking mother | Socioeconomic factors and family | 41.2 | 0.4195 | (0.3338, 0.5052) | 0.0006 |
| Gambling | Any gambling | Mental health and wellbeing | 1.79 | -0.0959 | (-0.1642,  -0.0275) | 0.0001 |
| ▪Mean_Accelerometer | Total minutes in MVPA: 1min epochs where ENMO > 100mg | Lifestyle and peer relationships | 55.37 | -0.1461 | (-0.1742,  -0.118) | 0.0005 |
| PotentialSexMin | Potential sexual minority | Other | 2.21 | 0.4113 | (0.3199, 0.5026) | 0 |
| Religion | Religious (any religion) | Socioeconomic factors and family | 0 | -0.0413 | (-0.0844, 0.0019) | 0.0003 |

AUDIT: Alcohol Use Disorders Identification Test; CGT: Cambridge Gambling Task; CM: Cohort Member; SDQ: Strengths and difficulties questionnaire

The overall (average) percentage of missing values was 6.12 %.

# Supplementary Table 2. All predictors, univariate coefficients and multivariate feature importances of the KiGGS Cohort

A detailed description of the instruments is given in:

Mauz E, Lange M, Houben R, et al. Cohort profile: KiGGS cohort longitudinal study on the health of children, adolescents and young adults in Germany. Int J Epidemiol 2020; 49: 375–375k.

▪ Variable is part of the physical inactivity assessment

▪ Variable is part of the screen time assessment

▪ Variable is part of the peer problems assessment

| **Variable Code** | **Variable Description** | **Domain assignment** | **Percent missing values** | **Univariate Association** | **Univariate Association CI** | **Multivariate Feature Importance** |
| --- | --- | --- | --- | --- | --- | --- |
| Age | Age | Other | 0 | 0.0288 | (-0.0359, 0.0935) | 0.0003 |
| AKalk | Ever drunk alcohol | Lifestyle and peer relationships | 3.63 | 0.1739 | (-0.0166, 0.3643) | 0.0016 |
| bik |  | Socioeconomic factors and family | 0 |  |  | 0.0239 |
| bik_0.0 | Lives in place with <2000 inhabitants | Socioeconomic factors and family |  | -0.4939 | (-0.9109,  -0.0768) |  |
| bik_1.0 | Lives in place with 2000 to 5000 inhabitants | Socioeconomic factors and family |  | -0.2204 | (-0.5487, 0.108) |  |
| bik_2.0 | Lives in place with 5000 to 20000 inhabitants | Socioeconomic factors and family |  | -0.2129 | (-0.435, 0.0092) |  |
| bik_3.0 | Lives in place with 20000 to 50000 inhabitants | Socioeconomic factors and family |  | 0.3926 | (0.1853, 0.6) |  |
| bik_4.0 | Lives in place with 50000 to 100000 inhabitants, surrounding area | Socioeconomic factors and family |  | -0.0355 | (-0.2985, 0.2275) |  |
| bik_5.0 | Lives in place with 50000 to 100000 inhabitants, core city | Socioeconomic factors and family |  | 0.4209 | (-0.0634, 0.9052) |  |
| bik_6.0 | Lives in place with 100000 to 500000 inhabitants, surrounding area | Socioeconomic factors and family |  | -0.0792 | (-0.2364, 0.078) |  |
| bik_7.0 | Lives in place with 100000 to 500000 inhabitants, core city | Socioeconomic factors and family |  | 0.2825 | (0.1079, 0.4571) |  |
| bik_8.0 | Lives in place with >500000 inhabitants, surrounding area | Socioeconomic factors and family |  | 0.0759 | (-0.135, 0.2869) |  |
| bik_9.0 | Lives in place with >500000 inhabitants, core city | Socioeconomic factors and family |  | -0.2165 | (-0.3726,  -0.0604) |  |
| BLEalleinmv | Burden due to living alone, loneliness (parent report) | Socioeconomic factors and family | 3.63 | 0.1778 | (0.1234, 0.2323) | 0.0021 |
| BLEalltagmv | Stress in everyday life as a whole (parent report) | Socioeconomic factors and family | 0.58 | 0.0301 | (-0.0339, 0.0941) | 0.0018 |
| BLEanerkenmv | Burden due to low recognition for housework/family work (parent report) | Socioeconomic factors and family | 0.58 | 0.0862 | (0.0259, 0.1464) | 0.0032 |
| BLEbehkindmv | Burden due to behind. or chron. ill child (parent report) | Socioeconomic factors and family | 0.58 | 0.0782 | (0.0104, 0.1459) | 0.0013 |
| BLEberufmv | Burden due to occupational situation or unemployment (parent report) | Socioeconomic factors and family | 1.16 | 0.0591 | (-0.0025, 0.1206) | 0.0006 |
| BLEfamberufmv | Burden due to problems in reconciling family and work (parent report) | Socioeconomic factors and family | 0.5 | 0.0676 | (0.0046, 0.1306) | 0.0022 |
| BLEfammv | Burden due to constant family involvement (parent report) | Socioeconomic factors and family | 0.5 | -0.0453 | (-0.1091, 0.0185) | 0.0017 |
| BLEfinanzmv | Burden due to financial worries (parent report) | Socioeconomic factors and family | 0.41 | 0.1223 | (0.0616, 0.183) | 0.0066 |
| BLEharbeitmv | Burden due to housework (parent report) | Socioeconomic factors and family | 0.41 | 0.0021 | (-0.0617, 0.0659) | 0.0007 |
| BLEkinder1mv | Burden due to sole responsibility for raising children (parent report) | Socioeconomic factors and family | 0.41 | 0.1431 | (0.0805, 0.2058) | 0.0056 |
| BLEkinder2mv | Burden due to parenting problems/conflicts with children (parent report) | Socioeconomic factors and family | 0.5 | 0.0923 | (0.0284, 0.1562) | 0.0016 |
| BLEpartmv | Burden due to conflicts with (ex)partner (parent report) | Socioeconomic factors and family | 0.74 | 0.1619 | (0.1008, 0.2231) | 0.0026 |
| BLEpflegemvC | Burden due to family members in need of care/seriously ill (parent report) | Socioeconomic factors and family | 0.58 | 0.0192 | (-0.0465, 0.085) | 0.0022 |
| BLEprobfammv | Burden due to other family members (parent report) | Socioeconomic factors and family | 0.5 | 0.1276 | (0.0685, 0.1867) | 0.0023 |
| bula |  | Socioeconomic factors and family | 0 |  |  | 0.1076 |
| bula_0.0 | State: Schleswig-Holstein | Socioeconomic factors and family |  | 1.1549 | (0.8127, 1.4971) |  |
| bula_1.0 | State: Hamburg | Socioeconomic factors and family |  | -0.8499 | (-1.3781,  -0.3217) |  |
| bula_10.0 | State: Berlin | Socioeconomic factors and family |  | 0.0516 | (-0.2876, 0.3907) |  |
| bula_11.0 | State: Brandenburg | Socioeconomic factors and family |  | 0.2417 | (-0.1682, 0.6517) |  |
| bula_12.0 | State: Mecklenburg-Vorpommern | Socioeconomic factors and family |  | -0.0286 | (-0.5379, 0.4808) |  |
| bula_13.0 | State: Sachsen | Socioeconomic factors and family |  | -0.2402 | (-0.5732, 0.0928) |  |
| bula_14.0 | State: Sachsen-Anhalt | Socioeconomic factors and family |  | -0.1695 | (-0.5755, 0.2366) |  |
| bula_15.0 | State: Thüringen | Socioeconomic factors and family |  | 0.0469 | (-0.4056, 0.4994) |  |
| bula_2.0 | State: Niedersachsen | Socioeconomic factors and family |  | -0.0425 | (-0.2499, 0.1649) |  |
| bula_3.0 | State: Bremen | Socioeconomic factors and family |  | -0.5047 | (-1.1548, 0.1455) |  |
| bula_4.0 | State: Nordrhein-Westfalen | Socioeconomic factors and family |  | 0.2682 | (0.1175, 0.419) |  |
| bula_5.0 | State: Hessen | Socioeconomic factors and family |  | -0.0791 | (-0.3354, 0.1771) |  |
| bula_6.0 | State: Rheinland-Pfalz | Socioeconomic factors and family |  | -0.0788 | (-0.362, 0.2044) |  |
| bula_7.0 | State: Baden-Württemberg | Socioeconomic factors and family |  | -0.0794 | (-0.2556, 0.0967) |  |
| bula_8.0 | State: Bayern | Socioeconomic factors and family |  | -0.3189 | (-0.4872,  -0.1506) |  |
| bula_9.0 | State: Saarland | Socioeconomic factors and family |  | 0.3768 | (-0.2184, 0.9719) |  |
| ENgem | Number of vegetables eaten daily | Lifestyle and peer relationships | 3.63 | -0.034 | (-0.0991, 0.0312) | 0.0027 |
| ENsuess | Number of sweets eaten daily | Lifestyle and peer relationships | 3.63 | -0.0083 | (-0.0709, 0.0543) | 0.0012 |
| GEopf1B | Victim of violence in last 12 months | Other | 3.63 | 0.3159 | (0.015, 0.6168) | 0.0018 |
| GEtat1B | Violence perpetrated in last 12 months | Other | 3.71 | -0.0998 | (-0.3622, 0.1627) | 0 |
| GZEmehm1 | General state of health (parent report) | Physical health | 0.33 | 0.1782 | (0.1171, 0.2394) | 0.0032 |
| GZmehm1 | General state of health (self report) | Physical health | 3.55 | 0.1086 | (0.0468, 0.1704) | 0.0018 |
| GZmehm2 | Impairment due to illness | Physical health | 13.12 | -0.0718 | (-0.1328,  -0.0107) | 0.0003 |
| GZmehm3B | Existing chronic diseases | Physical health | 4.37 | -0.0411 | (-0.2287, 0.1465) | 0 |
| IAarztzE | Number of contacts with any registered physician in last 12 months | Physical health | 4.62 | 0.0632 | (0.0012, 0.1252) | 0.0089 |
| IAarztzE16 | Number of contacts with psychologist in last 12 months | Mental health and wellbeing | 0.74 | 0.1415 | (0.0819, 0.2011) | 0.0006 |
| IAarztzE7 | Number of contacts with neurologist or psychiatrist in last 12 months | Mental health and wellbeing | 0.74 | 0.0343 | (-0.0339, 0.1024) | 0 |
| IAkhs | Inpatient in hospital in last 12 months | Physical health | 0.66 | -0.011 | (-0.2788, 0.2568) | 0 |
| ▪ KAempfzB | Number of days physically active last seven days | Lifestyle and peer relationships | 4.21 | -0.0948 | (-0.1567,  -0.0329) | 0.0075 |
| ▪ KAspodauz | Total hours sports per week | Lifestyle and peer relationships | 17 | 0.0008 | (-0.0669, 0.0686) | 0.018 |
| KAverein | Participation in club sports | Lifestyle and peer relationships | 4.21 | -0.2768 | (-0.4165,  -0.137) | 0.0032 |
| KHab | Ever had bronchial asthma (physician diagnosis) | Physical health | 1.98 | 0.2224 | (-0.0182, 0.4631) | 0.0041 |
| KHalgi1B | Ever allergic rhinitis (physician diagnosis) | Physical health | 1.98 | -0.1089 | (-0.2873, 0.0694) | 0 |
| KHalgi2 | Ever contact dermatitis (physician diagnosis) | Physical health | 1.57 | 0.0866 | (-0.1734, 0.3466) | 0.0007 |
| KHalgi3B | Ever neurodermatitis (physician diagnosis) | Physical health | 1.98 | 0.1492 | (-0.0167, 0.315) | 0.0001 |
| KHbron | Ever obstructive/spastic. Bronchitis (physician diagnosis) | Physical health | 1.98 | 0.1168 | (-0.0368, 0.2705) | 0.0005 |
| KJEgebgewi | Birth weight [g] | Other | 1.07 | -0.0077 | (-0.0717, 0.0564) | 0.0183 |
| KJEnote1z | School grade: Mathematics | Psychological and cognitive testing | 3.14 | 0.066 | (0.003, 0.129) | 0.0022 |
| KJEnote2z | School grade: German | Psychological and cognitive testing | 3.38 | 0.0436 | (-0.0214, 0.1085) | 0.0018 |
| KJEsect | Born by cesarean section | Other | 51.73 | -0.2217 | (-0.4369,  -0.0066) | 0.0001 |
| KSEsubj | Dissatisfaction with body weight (parent report) | Mental health and wellbeing | 0.33 | 0.0936 | (0.0304, 0.1569) | 0.0074 |
| KSsubj | Dissatisfaction with body weight (self report) | Mental health and wellbeing | 3.63 | 0.1269 | (0.0649, 0.189) | 0.0008 |
| LQkids1 | Quality of life: Well-being | Mental health and wellbeing | 3.55 | -0.0858 | (-0.1488,  -0.0229) | 0.0041 |
| LQkids10B | Quality of life: Concentration | Mental health and wellbeing | 3.63 | 0.0015 | (-0.061, 0.064) | 0.0006 |
| LQkids2B | Quality of life: Energy | Mental health and wellbeing | 3.55 | -0.0469 | (-0.1115, 0.0177) | 0.0049 |
| LQkids3B | Quality of life: Sad | Mental health and wellbeing | 3.63 | 0.1602 | (0.0972, 0.2232) | 0.0072 |
| LQkids4B | Quality of life: Lonely | Mental health and wellbeing | 3.63 | 0.1353 | (0.0747, 0.1959) | 0.001 |
| LQkids5B | Quality of life: Time | Mental health and wellbeing | 3.55 | 0.0566 | (-0.0062, 0.1194) | 0.0187 |
| LQkids6B | Quality of life: Free time | Mental health and wellbeing | 3.63 | -0.0127 | (-0.0757, 0.0504) | 0.0011 |
| LQkids7B | Quality of life: Treated fairly | Mental health and wellbeing | 3.63 | -0.0382 | (-0.0973, 0.021) | 0.0006 |
| LQkids8B | Quality of life: Fun with friends | Mental health and wellbeing | 3.63 | -0.0226 | (-0.0831, 0.0379) | 0 |
| LQkids9 | Quality of life: School | Mental health and wellbeing | 3.71 | -0.1495 | (-0.2092,  -0.0898) | 0.0047 |
| MImigrant | Migrant status | Socioeconomic factors and family | 0.08 | 0.4205 | (0.2536, 0.5875) | 0.0133 |
| ▪ MNfern | Television/video watching (hrs./day) | Lifestyle and peer relationships | 3.63 | 0.0295 | (-0.0348, 0.0937) | 0.004 |
| ▪ MNhandy | Cell phone use (hrs./day) | Lifestyle and peer relationships | 3.63 | 0.0939 | (0.0281, 0.1597) | 0.0016 |
| ▪ MNkons | Game console (hrs./day) | Lifestyle and peer relationships | 3.8 | -0.0384 | (-0.1045, 0.0277) | 0.0037 |
| ▪ MNpc | Computer/Internet (hrs/day) | Lifestyle and peer relationships | 3.71 | 0.0729 | (0.0101, 0.1358) | 0.0033 |
| PAbmi | BMI | Physical health | 0.41 | 0.0944 | (0.0323, 0.1565) | 0.0353 |
| PAEaufortB | Single parent | Socioeconomic factors and family | 0.58 | -0.2028 | (-0.3988,  -0.0067) | 0.0003 |
| PAEgeschwhhz | Number of siblings in household | Socioeconomic factors and family | 1.24 | 0.0299 | (-0.0327, 0.0925) | 0.0061 |
| PAEpos | Position in sibling order | Socioeconomic factors and family | 15.26 | 0.047 | (-0.0175, 0.1116) | 0.0056 |
| PKadhs | Ever ADHD diagnosed | Mental health and wellbeing | 4.7 | 0.0472 | (-0.2215, 0.3159) | 0.0002 |
| PKEkhm | Former mental illness (mother) | Socioeconomic factors and family | 1.16 | 0.3836 | (0.0872, 0.68) | 0.0027 |
| PKEkhm_zz | Current mental illness (mother) | Socioeconomic factors and family | 13.04 | 1.067 | (0.7587, 1.3753) | 0.0002 |
| PKEkhv | Former mental illness (father) | Socioeconomic factors and family | 11.06 | -0.05 | (-0.5483, 0.4482) | 0.0156 |
| PKEkhv_zz | Current mental illness (father) | Socioeconomic factors and family | 0.91 | 0.4798 | (-0.1189, 1.0786) | 0.0011 |
| PKscoff | SCOFF: Possible eating disorder^1^ | Mental health and wellbeing | 5.53 | 0.3276 | (0.1463, 0.509) | 0.0006 |
| RCErauch2m | Current smoking (mother) | Socioeconomic factors and family | 0.83 | 0.2788 | (0.1341, 0.4234) | 0.0007 |
| RCErauch2v | Current smoking (father) | Socioeconomic factors and family | 11.14 | 0.2228 | (0.0801, 0.3654) | 0 |
| RCEschw | Smoking during pregnancy | Socioeconomic factors and family | 0 | 0.1479 | (-0.0202, 0.316) | 0 |
| RCEstill | Smoking during breastfeeding | Socioeconomic factors and family | 0 | 0.0694 | (-0.1514, 0.2902) | 0.0007 |
| SDEses_ber | SES subscore: occupational status (parent report) | Socioeconomic factors and family | 0.5 | -0.183 | (-0.2415,  -0.1245) | 0.0337 |
| SDEses_bild | SES subscore: education/training (parent report) | Socioeconomic factors and family | 0.41 | -0.1851 | (-0.2458,  -0.1244) | 0.0375 |
| SDEses_eink | SES subscore: income (parent report) | Socioeconomic factors and family | 0.5 | -0.1571 | (-0.2178,  -0.0964) | 0.0158 |
| SDgeld2 | Has own income | Other | 3.55 | -0.2069 | (-0.3678,  -0.046) | 0.0044 |
| sex | Sex (male compared to female) | Other | 0 | -0.5462 | (-0.6692,  -0.4231) | 0.1439 |
| SFfamzus | Family cohesion scale score^2^ | Socioeconomic factors and family | 3.55 | -0.0492 | (-0.1112, 0.0128) | 0.0051 |
| SFprs | Personal ressources scale score^3^ | Socioeconomic factors and family | 3.55 | -0.087 | (-0.1503,  -0.0237) | 0.0122 |
| ▪ SFsss | Social support scale score^4^ | Socioeconomic factors and family | 3.55 | -0.0277 | (-0.092, 0.0367) | 0.0054 |
| SZarm_k | Arm pain last 3 months | Physical health | 3.63 | 0.0352 | (-0.1408, 0.2111) | 0 |
| SZbauch_k | Abdominal pain last 3 months | Physical health | 3.55 | 0.1827 | (0.0531, 0.3124) | 0.0021 |
| SZbein_k | Leg pain last 3 months | Physical health | 3.55 | -0.0223 | (-0.1572, 0.1127) | 0 |
| SZkopf_k | Headache last 3 months | Physical health | 3.71 | 0.0638 | (-0.0664, 0.1941) | 0.0018 |
| SZmens_k | Period pain last 3 months | Physical health | 53.63 | 0.6402 | (0.3987, 0.8817) | 0.0185 |
| SZrueck_k | Back pain last 3 months | Physical health | 3.55 | 0.0134 | (-0.1354, 0.1623) | 0.0005 |
| SZsonst | Other pain last 3 months | Physical health | 3.63 | 0.3059 | (0.0266, 0.5851) | 0 |
| SZuleib_k | Abdominal pain last 3 months | Physical health | 3.8 | 0.4879 | (0.2921, 0.6837) | 0.0138 |
| UVarztC | Accident/poisoning with medical care last 12 months (parent report) | Physical health | 0.58 | 0.0835 | (-0.0871, 0.2542) | 0 |
| VAEemo | Sum score SQD emotional problems (parent report) | Mental health and wellbeing | 0.33 | 0.4747 | (0.422, 0.5273) | 0.6293 |
| VAEhyp | Sum score SQD inattention/hyperactivity (parent report) | Mental health and wellbeing | 0.33 | 0.1301 | (0.0646, 0.1956) | 0.0229 |
| VAemo | Sum score SQD emotional problems | Mental health and wellbeing | 3.55 | 0.2757 | (0.2151, 0.3364) | 0.0174 |
| ▪ VAEpeer | Sum score SQD peer problems (parent report) | Lifestyle and peer relationships | 0.33 | 0.202 | (0.1431, 0.2608) | 0.0072 |
| VAEpro | Sum score SQD prosocial behavior (parent report) | Mental health and wellbeing | 0.33 | 0.0273 | (-0.0332, 0.0878) | 0.006 |
| VAEverh | Sum score SQD behavioral problems (parent report) | Mental health and wellbeing | 0.33 | 0.0975 | (0.0359, 0.159) | 0.0018 |
| VAhyp | Sum score SQD inattention/hyperactivity | Mental health and wellbeing | 3.55 | 0.2066 | (0.1425, 0.2708) | 0.0372 |
| ▪ VApeer | Sum score SQD peer problems | Lifestyle and peer relationships | 3.55 | 0.1428 | (0.0794, 0.2062) | 0.0027 |
| VApro | Sum score SDQ prosocial behavior | Mental health and wellbeing | 3.55 | 0.109 | (0.0422, 0.1758) | 0.0257 |
| VAverh | Sum score SDQ behavioral problems | Mental health and wellbeing | 3.55 | 0.1228 | (0.0616, 0.1841) | 0.0119 |

The overall (average) percentage of missing values was 3.84 %.

1. **Morgan JF, Reid F, Lacey JH** (1999). The SCOFF questionnaire: assessment of a new screening tool for eating disorders. *British Medical Journal*, 319(7223), 1467-1468.
2. **Schneewind KA, Beckmann M, Hecht-Jackl, A** (1985). Sum score of four items from the Cohesion subscale of the Family Climate Scale (FCS), Das FK-Testsystem. Das Familienklima aus der Sichtweise der Eltern und der Kinder. Forschungsberichte aus dem Institutsbereich Persönlichkeitspsychologie und Psychodiagnostik
3. **Bettge S, Ravens-Sieberer U** (2003). Schutzfaktoren für die psychische Gesundheit von Kindern und Jugendlichen - empirische Ergebnisse zur Validierung eines Konzepts. *Gesundheitswesen*, 65, 167–172.
4. **Sherbourne CD, & Stewart AL** (1991). The MOS social support survey. *Social science & medicine*, 32(6), 705-714.

# Supplementary Table 3. Transparent reporting of a multivariable prediction model for individual prognosis or diagnosis (TRIPOD) checklist

| **Section/Topic** | **Item** |  | **Checklist Item** | **Page** |
| --- | --- | --- | --- | --- |
| **Title and abstract** | | | | |
| Title | 1 | D;V | Identify the study as developing and/or validating a multivariable prediction model, the target population, and the outcome to be predicted. | 1 |
| Abstract | 2 | D;V | Provide a summary of objectives, study design, setting, participants, sample size, predictors, outcome, statistical analysis, results, and conclusions. | 2 |
| **Introduction** | | | | |
| Background and objectives | 3a | D;V | Explain the medical context (including whether diagnostic or prognostic) and rationale for developing or validating the multivariable prediction model, including references to existing models. | 4 - 5 |
|  | 3b | D;V | Specify the objectives, including whether the study describes the development or validation of the model or both. | 4 - 5 |
| **Methods** | | | | |
| Source of data | 4a | D;V | Describe the study design or source of data (e.g., randomized trial, cohort, or registry data), separately for the development and validation data sets, if applicable. | 5 |
|  | 4b | D;V | Specify the key study dates, including start of accrual; end of accrual; and, if applicable, end of follow-up. | 5, 15 |
| Participants | 5a | D;V | Specify key elements of the study setting (e.g., primary care, secondary care, general population) including number and location of centres. | 5 |
|  | 5b | D;V | Describe eligibility criteria for participants. | 5, S2 |
|  | 5c | D;V | Give details of treatments received, if relevant. | n/a |
| Outcome | 6a | D;V | Clearly define the outcome that is predicted by the prediction model, including how and when assessed. | 5 |
|  | 6b | D;V | Report any actions to blind assessment of the outcome to be predicted. | n/a |
| Predictors | 7a | D;V | Clearly define all predictors used in developing or validating the multivariable prediction model, including how and when they were measured. | 5, S3 - S20 |
|  | 7b | D;V | Report any actions to blind assessment of predictors for the outcome and other predictors. | n/a |
| Sample size | 8 | D;V | Explain how the study size was arrived at. | S2 |
| Missing data | 9 | D;V | Describe how missing data were handled (e.g., complete-case analysis, single imputation, multiple imputation) with details of any imputation method. | 6, 16 - 17 |
| Statistical analysis methods | 10a | D | Describe how predictors were handled in the analyses. | 6 - 7, 16 - 17 |
|  | 10b | D | Specify type of model, all model-building procedures (including any predictor selection), and method for internal validation. | 6 - 7, 16 - 17 |
|  | 10c | V | For validation, describe how the predictions were calculated. | 6 - 7, 16 - 17 |
|  | 10d | D;V | Specify all measures used to assess model performance and, if relevant, to compare multiple models. | 6, 8, 15, S21 |
|  | 10e | V | Describe any model updating (e.g., recalibration) arising from the validation, if done. | n/a |
| Risk groups | 11 | D;V | Provide details on how risk groups were created, if done. | n/a |
| Development vs. validation | 12 | V | For validation, identify any differences from the development data in setting, eligibility criteria, outcome, and predictors. | n/a |
| **Results** | | | | |
| Participants | 13a | D;V | Describe the flow of participants through the study, including the number of participants with and without the outcome and, if applicable, a summary of the follow-up time. A diagram may be helpful. | S2 |
|  | 13b | D;V | Describe the characteristics of the participants (basic demographics, clinical features, available predictors), including the number of participants with missing data for predictors and outcome. | 15, S3 - S20 |
|  | 13c | V | For validation, show a comparison with the development data of the distribution of important variables (demographics, predictors and outcome). | n/a |
| Model development | 14a | D | Specify the number of participants and outcome events in each analysis. | 15 |
|  | 14b | D | If done, report the unadjusted association between each candidate predictor and outcome. | 16, S3 - S20 |
| Model specification | 15a | D | Present the full prediction model to allow predictions for individuals (i.e., all regression coefficients, and model intercept or baseline survival at a given time point). | n/a |
|  | 15b | D | Explain how to the use the prediction model. | n/a |
| Model performance | 16 | D;V | Report performance measures (with CIs) for the prediction model. | 15, S21 |
| Model-updating | 17 | V | If done, report the results from any model updating (i.e., model specification, model performance). | n/a |
| **Discussion** | | | | |
| Limitations | 18 | D;V | Discuss any limitations of the study (such as nonrepresentative sample, few events per predictor, missing data). | 10 |
| Interpretation | 19a | V | For validation, discuss the results with reference to performance in the development data, and any other validation data. | n/a |
|  | 19b | D;V | Give an overall interpretation of the results, considering objectives, limitations, results from similar studies, and other relevant evidence. | 9 - 11 |
| Implications | 20 | D;V | Discuss the potential clinical use of the model and implications for future research. | 10 |
| **Other information** | | | | |
| Supplementary information | 21 | D;V | Provide information about the availability of supplementary resources, such as study protocol, Web calculator, and data sets. | 7 |
| Funding | 22 | D;V | Give the source of funding and the role of the funders for the present study. | 7 |

*Items relevant only to the development of a prediction model are denoted by D, items relating solely to a validation of a prediction model are denoted by V, and items relating to both are denoted D;V.
